# Insights into the structure and dynamics of lysyl oxidase propeptide, a flexible protein with numerous partners

**DOI:** 10.1038/s41598-018-30190-6

**Published:** 2018-08-06

**Authors:** Sylvain D. Vallet, Adriana E. Miele, Urszula Uciechowska-Kaczmarzyk, Adam Liwo, Bertrand Duclos, Sergey A. Samsonov, Sylvie Ricard-Blum

**Affiliations:** 1 Univ Lyon, University Claude Bernard Lyon 1, CNRS, INSA Lyon, CPE, Institute of Molecular and Supramolecular Chemistry and Biochemistry, UMR 5246, F-69622 Villeurbanne cedex, France; 2 Laboratory of Molecular Modeling, Department of Theoretical Chemistry, Faculty of Chemistry, University of Gdansk, Wita Stwosza 63, 80-308 Gdańsk, Poland

## Abstract

Lysyl oxidase (LOX) catalyzes the oxidative deamination of lysine and hydroxylysine residues in collagens and elastin, which is the first step of the cross-linking of these extracellular matrix proteins. It is secreted as a proenzyme activated by bone morphogenetic protein-1, which releases the LOX catalytic domain and its bioactive N-terminal propeptide. We characterized the recombinant human propeptide by circular dichroism, dynamic light scattering, and small-angle X-ray scattering (SAXS), and showed that it is elongated, monomeric, disordered and flexible (D_max_: 11.7 nm, R_g_: 3.7 nm). We generated 3D models of the propeptide by coarse-grained molecular dynamics simulations restrained by SAXS data, which were used for docking experiments. Furthermore, we have identified 17 new binding partners of the propeptide by label-free assays. They include four glycosaminoglycans (hyaluronan, chondroitin, dermatan and heparan sulfate), collagen I, cross-linking and proteolytic enzymes (lysyl oxidase-like 2, transglutaminase-2, matrix metalloproteinase-2), a proteoglycan (fibromodulin), one growth factor (Epidermal Growth Factor, EGF), and one membrane protein (tumor endothelial marker-8). This suggests new roles for the propeptide in EGF signaling pathway.

## Introduction

Lysyl oxidase (LOX), a copper amine oxidase, catalyzes the oxidative deamination of lysine and hydroxylysine residues in collagens and elastin, which is the first step of the covalent cross-linking of these extracellular matrix (ECM) proteins^1^. LOX stimulates angiogenesis *in vivo*^2^, and in cancer, where it contributes to the formation of pre-metastatic “niche”, and to metastasis^3–5^. LOX is secreted as a proenzyme. It is activated by bone morphogenetic protein 1 (BMP-1), which releases the mature catalytic domain of LOX and its N-terminal propeptide. The propeptide of lysyl oxidase (LOX-PP) has various biological activities of its own^6^, and might thus be considered as a matricryptin, *i*.*e*. a bioactive ECM fragment exhibiting other functions than its parent molecule^7,8^. LOX-PP is required for the exit of proLOX from the endoplasmic reticulum^9^, and for the deposition of LOX onto elastic fibers^10^. It induces phenotypic reversion of ras-transformed cells^11^, and inhibits cell signaling and proliferation of smooth muscle cells and osteoblasts^12,13^. The inhibition of FGF-2 signaling by LOX-PP in NIH 3T3-L1 cells contributes to its proadipogenic effect^14^. LOX-PP inhibits the growth of prostate cancer cells by interacting with DNA repair proteins^15^. Indeed, LOX-PP has been found in the nucleus of several cell types^16,17^, and colocalizes with the microtubule network in osteoblastic cells^17^. LOX-PP interacts with intracellular or nuclear proteins, namely HSP70 and c-Raf, CIN85, UXT^18–20^ and with the intracellular phosphatase domains of receptor-type protein tyrosine phosphatase kappa^21^. The major uptake pathway of recombinant LOX-PP is phosphatidylinositol-4,5-bisphosphate 3-kinase (PI3K)-dependent micropinocytosis, while a secondary pathway depends on dynamin and caveolae^22^. LOX-PP also interacts with two extracellular proteins, elastin^10^ and fibronectin^23^, and contains cleavage sites for matrix metalloproteinases (MMPs) 2 and 10^24,25^. We have recently shown that LOX-PP binds to heparin^26^ (HP).

Numerous cell studies, including those cited above, were performed with the recombinant rat propeptide, which contains both N- and O-linked carbohydrates^27^, and has been shown to be highly disordered by circular dichroism (CD). However, no structural data are available for the human propeptide so far. We report here the characterization of recombinant human LOX-PP by circular dichroism, dynamic light scattering (DLS), and small-angle X-ray scattering (SAXS), which showed that human LOX-PP is an elongated, monomeric, flexible, protein enriched in intrinsic disorder. We generated 3D models of LOX-PP by coarse-grained molecular dynamics (MD) simulations restrained by the distance distribution derived from SAXS measurements. LOX-PP being a heparin-binding protein^26^, we used these models for docking experiments with a hexasaccharide of heparin to localize the heparin-binding site. The analysis of the docked structures by all-atomic molecular dynamics and free energy showed that the hexasaccharide may interact with the propeptide *via* two regions. Furthermore, we have explored the ability of LOX-PP to interact with the ECM, and we have identified 17 new partners of LOX-PP, including four glycosaminoglycans (GAGs, chondroitin sulfate, dermatan sulfate, heparan sulfate, and hyaluronan), collagen I, cross-linking and proteolytic enzymes (lysyl oxidase-like 2, transglutaminase-2, matrix metalloproteinase-2), one proteoglycan (fibromodulin), one growth factor (Epidermal Growth Factor, EGF), and one membrane protein (Tumor Endothelial Marker-8, TEM-8, also known as anthrax receptor-1). This suggests new roles for the propeptide in ECM assembly and cross-linking, cell-matrix adhesion, and in the regulation of EGF signaling pathways.

## Results

### Expression of recombinant human LOX-PP

**Figure 1:**
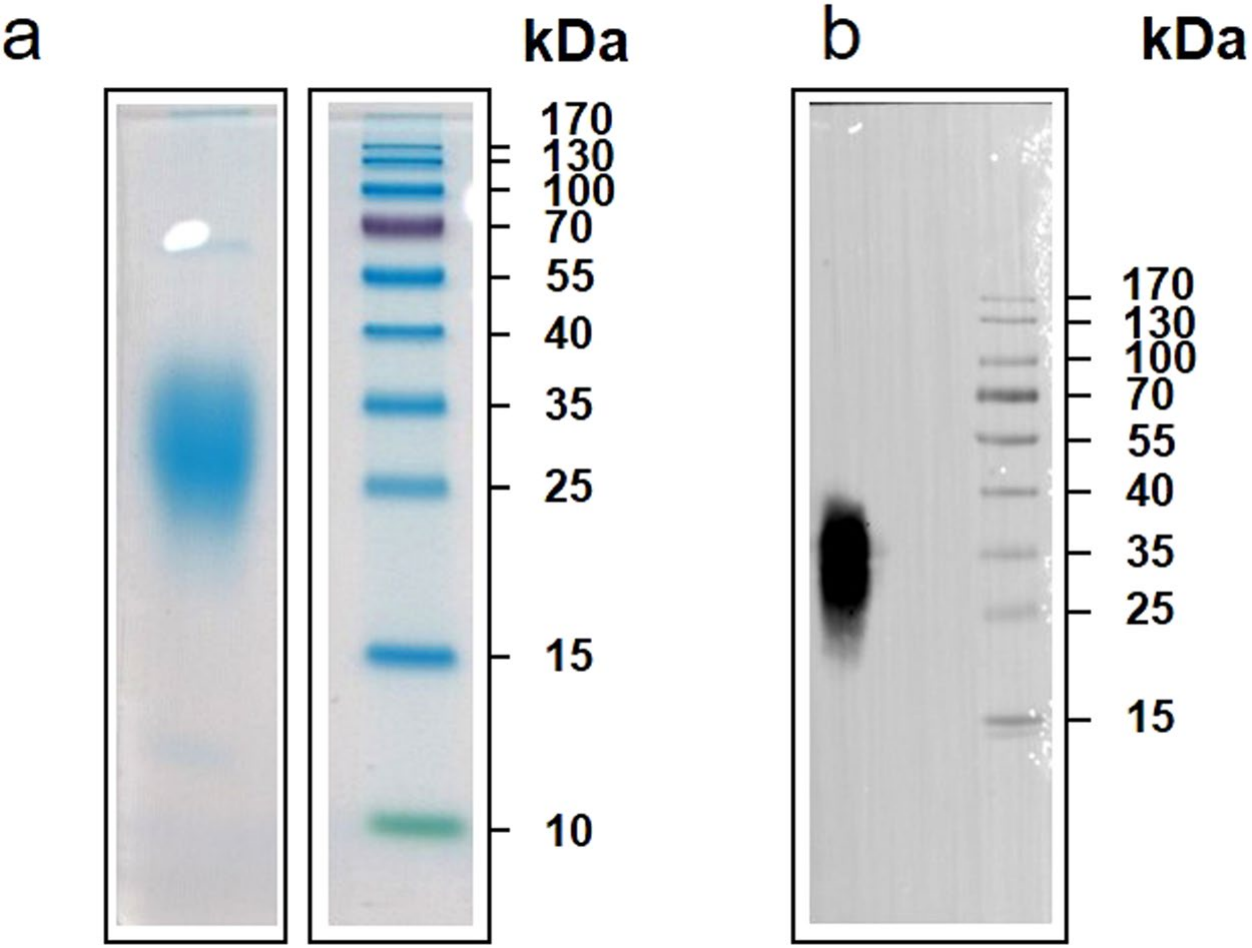
Analysis of the recombinant human propeptide of lysyl oxidase (LOX-PP) expressed in HEK293-EBNA cells. (**a**) SDS-PAGE (10% acrylamide running gel). (**b**) Western blot (anti-FLAG primary antibody^51^, detection by enhanced-chemiluminescence) of LOX-PP. The purification process was followed by SDS-PAGE and Western blot with a primary anti-FLAG antibody (F3165, Sigma-Aldrich^51^) and a secondary antibody conjugated to peroxidase (Bio-Rad, 172–1011). The gels were stained with EZBlue™ Gel Staining Reagent and were scanned with the Canon LiDE 210 scanner. For Western blots immunocomplexes were detected with SuperSignal West Pico Chemiluminescence Substrate (34080, Thermo Scientific) and visualized using the Fusion FX camera (Vilber-Lourmat) for three minutes with default settings. Pre-stained molecular weight markers were also visualized with the Fusion FX camera (Vilber-Lourmat), and superimposed on the membrane imaged by chemiluminescence with the Fusion Capt Advance software. Full-length gel and blot are presented. Lanes of the same gel were juxtaposed in (**a**), and consecutive lanes were presented for Western blot in (**b**).

Recombinant human LOX-PP expressed in Human Embryonic Kidney (HEK) 293 cells migrated with an apparent molecular weight of 30 kDa by sodium dodecyl sulfate – polyacrylamide gel electrophoresis (SDS-PAGE, Fig. 1a) although its theoretical molecular weight is 16.6 kDa. A single band was detected with an anti-FLAG antibody by Western blot (Fig. 1b). The apparent molecular weight was in agreement with the presence of glycosylation reported for the rat propeptide^27^. The deglycosylation of human LOX-PP by peptide N-glycosidase F (PNGase F), which removes N-linked oligosaccharides, resulted in a marked decrease in the apparent molecular weight of the human propeptide from 30 kDa to 17 kDa (Supplementary Fig. 1). This is consistent with the theoretical mass based on the amino acid sequence, and shows that N-glycans account for about 13 kDa of the molecular mass of human LOX-PP. The deglycosylated human propeptide migrated as a fuzzy and large band as previously observed for the deglycosylated rat protein^27^.

### Intrinsic disorder and secondary structure of human LOX-PP

**Figure 2:**
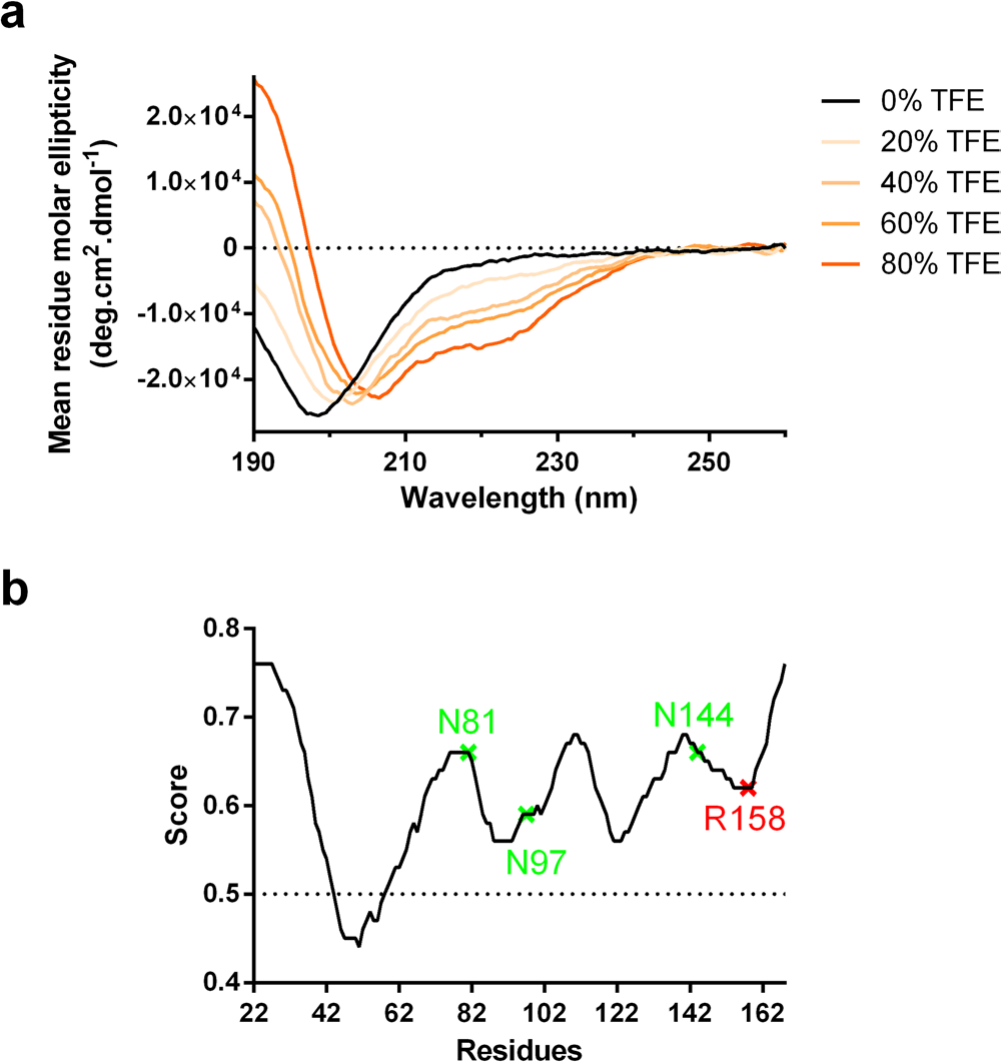
Secondary structure and intrinsic disorder of LOX-PP. (**a**) Averaged circular dichroism spectra (n = 5) of LOX-PP at 2 µM (60 µg/ml) in 10 mM potassium phosphate pH 7.4. Increasing concentrations of trifluoroethanol (TFE, 20, 40, 60, and 80%) were added to assess the ability of LOX-PP to fold into α helices. (**b**) Prediction with metaPrDOS of disordered residues in the sequence of LOX-PP (residues 22–168). The three glycosylated asparagine residues are represented in green and the arginine residue 158 in red, R158Q being a loss-of-function polymorphism described in breast cancer patients^42^.

Circular dichroism spectra of LOX-PP showed a single minimum near 200 nm, which is characteristic of an intrinsically disordered protein (IDP) (Fig. 2a). The content in secondary structure of LOX-PP was calculated by deconvoluting the spectra with CONTIN-LL. The content in α-helix and β-sheet was found to be 3.4% and 20.4% respectively, whereas the turn content was 11.8%, and the disorder 64.5%.

The amount of intrinsic disorder, predicted by metaPrDOS, was found to be 81% in the propeptide sequence alone and 86.4% in the context of the full-length LOX protein using the two-state prediction results with a given false positive of 5%. This suggests that the propeptide of LOX does not fold in presence of the catalytic domain of LOX. The three glycosylated asparagine residues and the arginine residue 158 (R158Q being a loss-of-function polymorphism) are in disordered regions, whereas the sequence encompassing residues 44 to 58 (QQIQWENNGQVFSLL) was predicted to be structured (Fig. 2b).

LOX-PP was analyzed in the presence of increasing concentrations of trifluoroethanol (TFE) (0–80%) to assess its ability to fold into α-helices (Fig. 2a). The content in α-helix of LOX-PP increased with TFE concentrations, ranging from 3.4% up to 25.2% at 80% TFE. The addition of full-length HP, a binding partner of LOX-PP, or of a HP hexasaccharide did not induce significant changes in the CD spectra of LOX-PP, either immediately after GAG addition or after a 60-min incubation (Supplementary Fig. 2).

### Analysis of LOX-PP by dynamic light scattering

**Figure 3:**
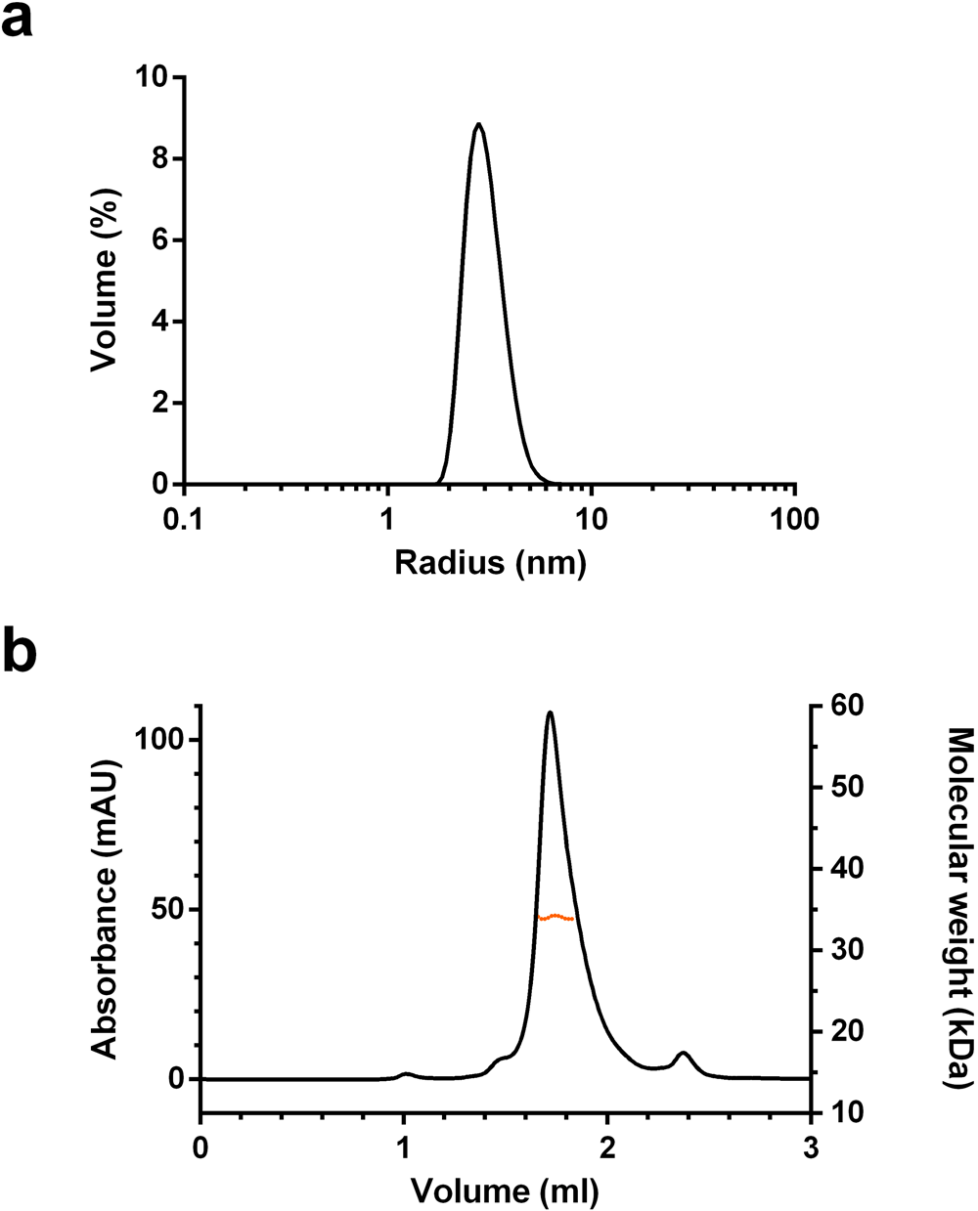
Analysis of LOX-PP by DLS and SEC-MALS. (**a**) Size distribution analysis of LOX-PP by dynamic light scattering in HBS at 20 °C (LOX-PP: 35 µM, 1.1 mg/ml, 4 × 15 curves were averaged). (**b**) LOX-PP (75 µM) in HBS was injected on a Superdex 200 Increase 5/150 GL column (GE Healthcare) for SEC-MALS analysis including UV absorbance at 280 nm, multi-angle light scattering at 690 nm and refractometry.

Four series of fifteen measurements were performed and gave a single peak with a polydispersity index of 0.817 ± 0.01. The hydrodynamic radius (R_h_) was calculated to be 3.05 ± 0.07 nm (Fig. 3a) with an estimated molecular weight of 55 ± 3 kDa, which might be due to the intrinsic disorder, flexibility and extensive glycosylation of LOX-PP.

### Analysis of LOX-PP by small-angle X-ray scattering

**Figure 4:**
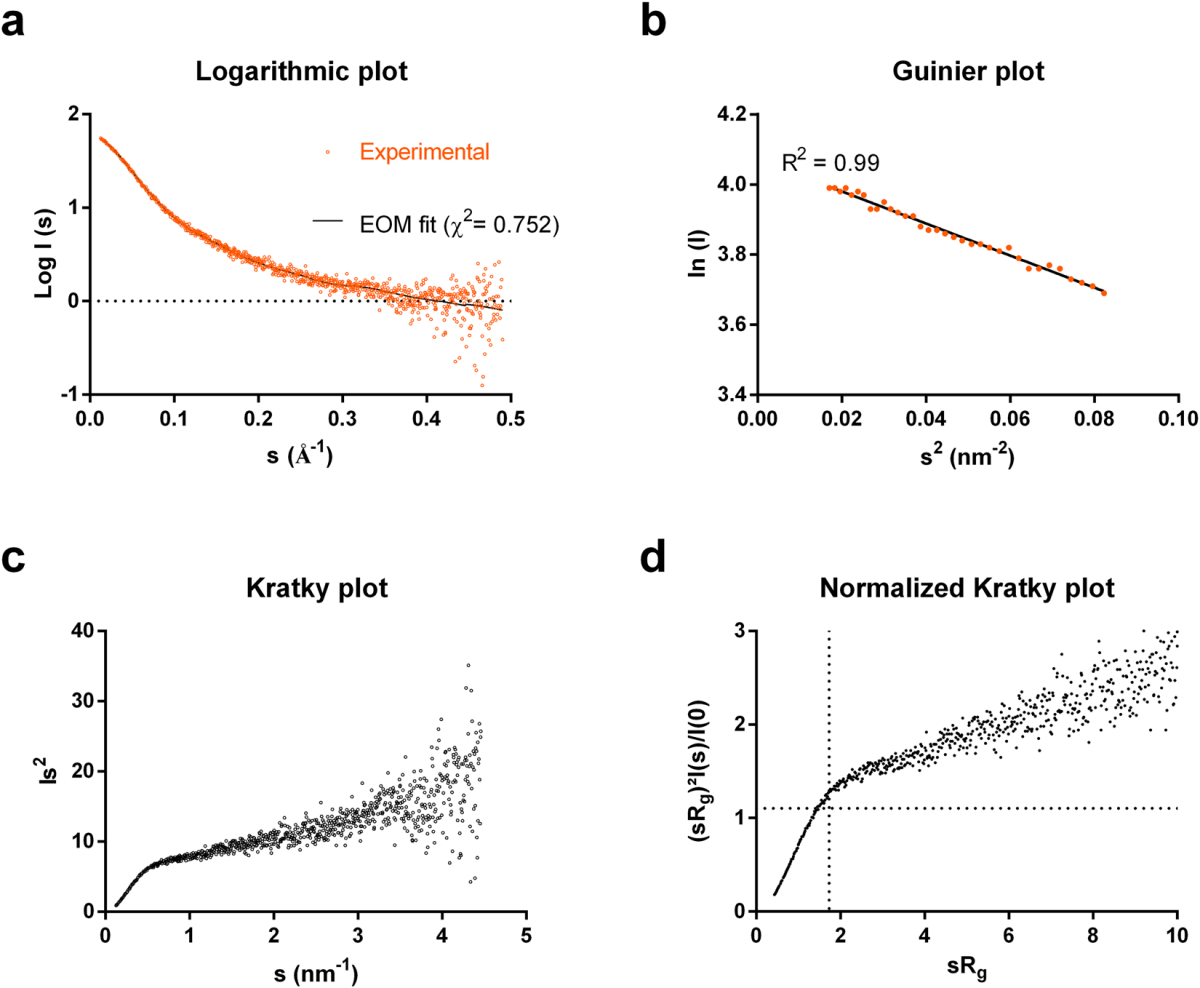
Small-angle X-ray scattering analysis of LOX-PP. (**a**) Logarithmic plot of experimental data. Fit of EOM 2.1 data to experimental SAXS data (χ² = 0.752). (**b**) Guinier plot of experimental data collected for LOX-PP in the range used to calculate the radius of gyration (R_g_ = 3.7 ± 0.04 nm) compared to the theoretical fit. Kratky plot (**c**) and Normalized Kratky plot of LOX-PP (**d**).

The shape of human LOX-PP in solution was studied on the BioSAXS BM29 beamline (European Synchrotron Radiation Facility, ESRF, Grenoble, France). 1D SAXS profiles showed no sign of aggregation as can be seen on the logarithmic plot (Fig. 4a). LOX-PP molecular weight calculated with autoprocessing framework EDNA ranged between 16 and 22 kDa, *a priori* consistent with a monomeric species and the presence of intrinsic disorder in LOX-PP as predicted by sequence analysis and confirmed by CD. The molecular weight of LOX-PP calculated with the SAXS Molecular Weight package (SAXSMoW^28^, http://saxs.ifsc.usp.br/, s_max_ value of 8/R_g_) was found to be 29.4 kDa. Size Exclusion Chromatography - Multi-Angle Light Scattering (SEC-MALS) analysis showed that glycosylated LOX-PP eluted as a single peak from a size-exclusion column with a molecular mass of 34.1 kDa (mass fraction: 97%, Fig. 3b). Both values are consistent with the apparent molecular weight calculated by SDS-PAGE (~ 30 kDa) and show that glycosylated LOX-PP is a monomer.

The radius of gyration (R_g_), determined with PRIMUS, was 3.7 ± 0.04 nm (Fig. 4b), which is of the same order of magnitude than the theoretical Rg (3.53 nm) calculated with Flory’s equation, and the hydrodynamic radius (3.05 nm).

The experimental R_g_/R_h_ ratio (1.2) was higher than 0.7, which indicates that LOX-PP behaves as a non-globular protein, and is likely to be extended^29^, which is consistent with the presence of intrinsic disorder in LOX-PP. Furthermore, the Kratky plot was indicative of a non-globular natively unfolded molecule (Fig. 4c), and the normalized Kratky plot confirmed that LOX-PP is indeed flexible (Fig. 4d). Experimental maximum dimension (D_max_) calculated from distance distribution function was 11.7 nm.

LOX-PP was also analyzed by SEC-SAXS on the SWING beamline (Small and Wide angle X-ray scattering, French National Synchrotron Facility SOLEIL, Saint-Aubin, France) to investigate the possible influence of trace amount of aggregation on the parameters calculated by SAXS. The calculated R_g_ values were 3.72 nm and 3.70 nm for SEC-SAXS and SAXS respectively, and those calculated for D_max_ were 11.8 nm and 11.7 nm for SEC-SAXS and SAXS, respectively (Supplementary Fig. 3). The values of R_g_ and D_max_ calculated for the propeptide by both SAXS and SEC-SAXS were thus nearly identical, showing that those calculated by SAXS were not affected by aggregation.

**Figure 5:**
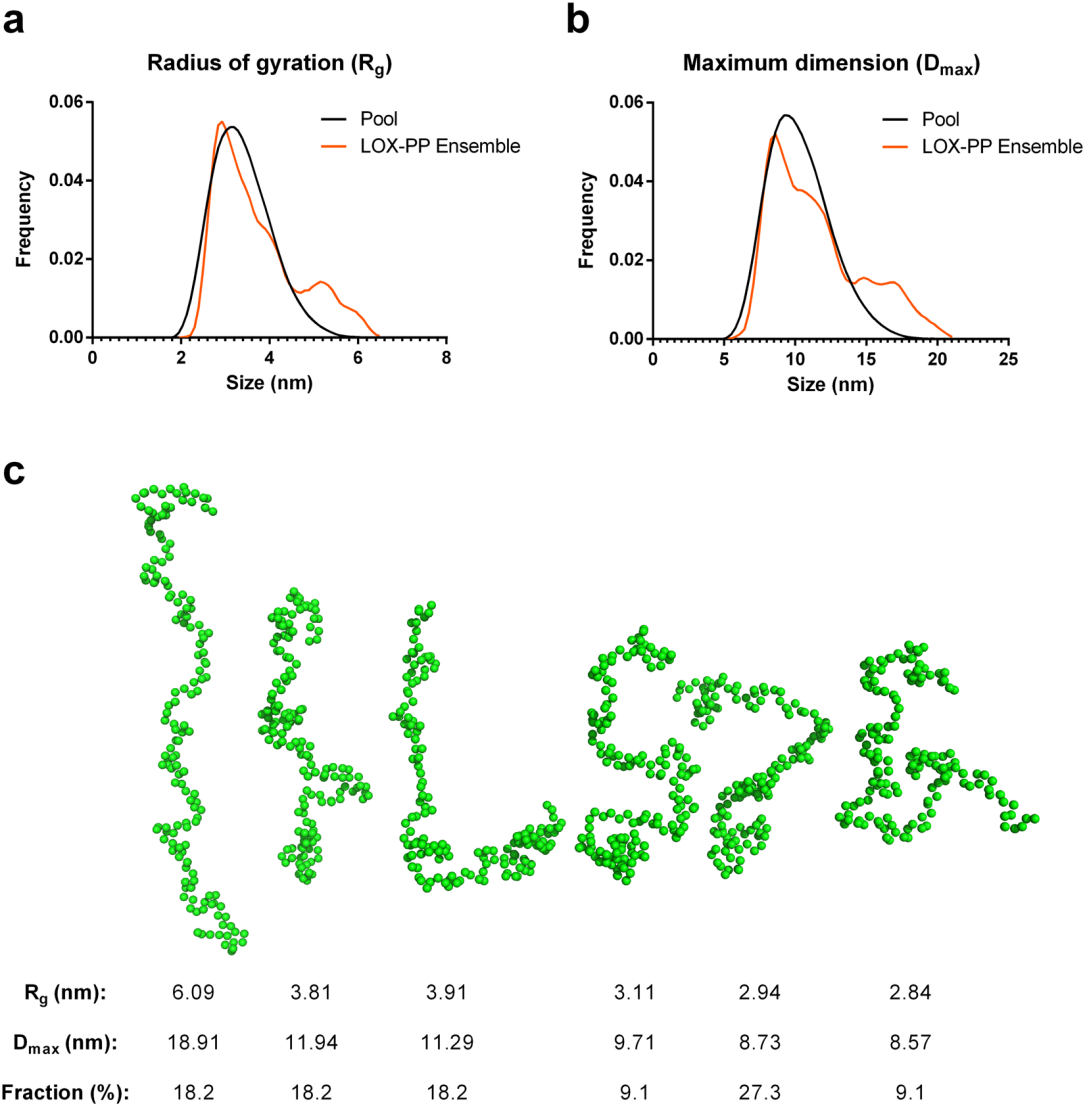
SAXS analysis by Ensemble Optimization Method (EOM). Distribution of R_g_ (**a**) and D_max_ (**b**) values for the generated pool and LOX-PP ensemble. (**c**) Beads models of LOX-PP built and selected by EOM to fit experimental data, presented by decreasing D_max_ value (Fraction %: fractions of occupancy).

Ensemble modelling (Ensemble Optimization Method, EOM 2.1) was used to identify the sub-ensembles of conformations of LOX-PP, which best fitted to SAXS experimental data. The best fit (χ^2^: 0.752, Fig. 4a) was obtained by a combination of six conformations of LOX-PP with an averaged R_g_ of 3.85 nm and D_max_ of 11.7 nm (Fig. 5a,b). R_flex_, which allows to quantify the difference between flexible and rigid systems^30^, was higher for the sub-ensemble of conformers coexisting in solution fitting to SAXS data than for the random pool of conformers covering the available conformational space (89.87% and 84.98% respectively), and R_σ_ was >1 (1.43). These results predicted the co-existence of several flexible conformers of LOX-PP in solution with a 2-fold variation in the D_max_ value, from 8.6 to 18.9 nm (Fig. 5c). However, these conformers correspond to the amino acid sequence of LOX-PP only, and not to its glycosylated form because glycans cannot be included in EOM modeling.

### Coarse-grained models of the propeptide of lysyl oxidase

**Figure 6:**
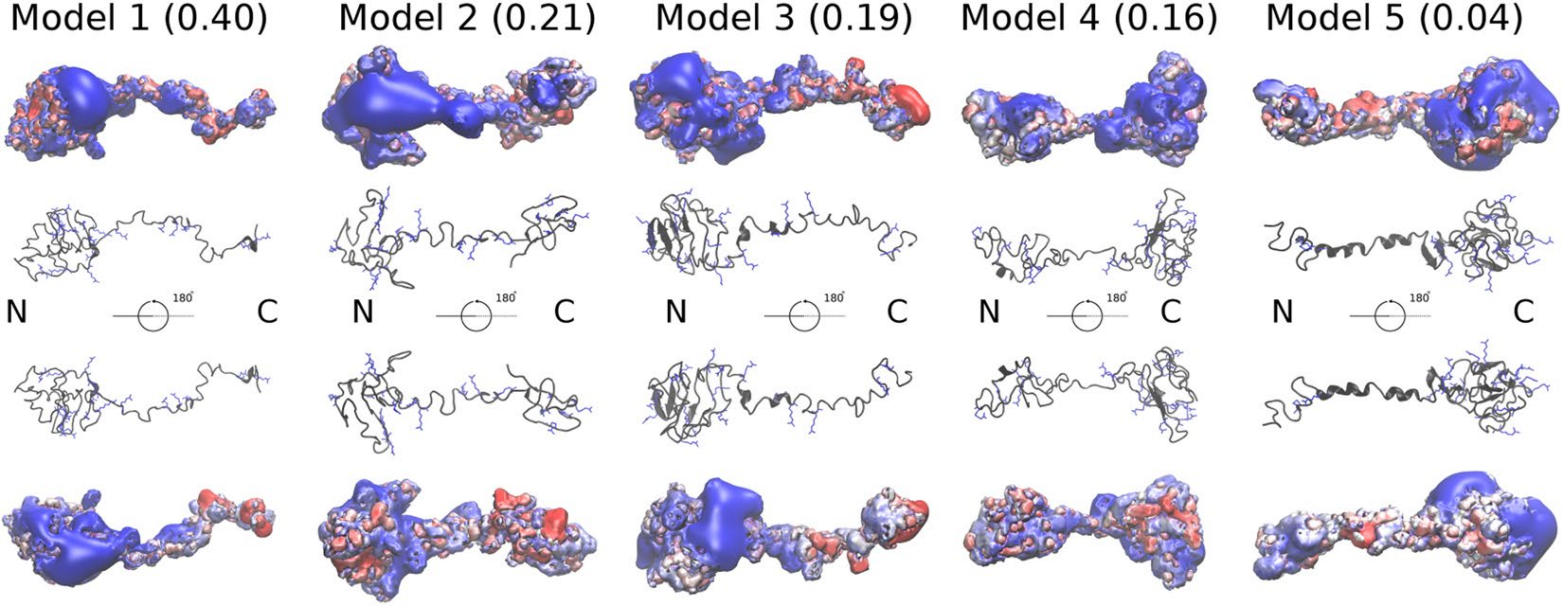
Models of LOX-PP obtained by coarse-grained MD simulations with UNRES. Their probability based on free energy calculations is indicated in brackets. Arginine residues are in blue sticks. The calculated electrostatic potential is represented by isosurfaces (in blue: positive electrostatic potential isosurface 3 kcal/mol·Å, in red: negative electrostatic potential isosurface −1 kcal/mol·Å). Different absolute isovalues were selected for negative and positive potentials due to the high positive charge of the protein (18 arginine residues out of 147).

Using the SAXS data-restrained UNRES/MREMD (United Residue Model of Proteins/ Multiple Replica Exchange Molecular Dynamics) modeling we obtained five three-dimensional (3D) models of LOX-PP (Fig. 6). The probability of model 1 (0.4) was significantly higher than those of the other models. Models 2, 3, and 4 had similar probabilities (0.21, 0.19, 0.16), while the probability of model 5 was very low (0.04). The N-termini of models 1 and 3 were folded and formed a globule, enriched in β-strands in model 3. Both N- and C-termini were folded in models 2 and 4, whereas in model 5 only the C-terminal part appeared to be globular. The central part of the propeptide was thus less structured than the termini, and part of it adopted an α-helical conformation in models 3 and 5 (Fig. 6). The radius of gyration of model 1 was 2.93 nm, which was in good agreement with SAXS and DLS data (~3 nm), while the radii of gyration of models 2–5 ranged from 2.56 to 2.74 nm (2.74 nm, model 2; 2.58 nm, model 3; 2.73 nm, model 4; 2.56 nm, model 5). The D_max_ value calculated for the five models (9.9, 8.1, 9.1, 7.9, and 8.6 nm) was lower than the experimental SAXS value (11.7 nm, Supplementary Fig. 4). It should be noted that the above models did not take into account the glycosylation of LOX-PP, whereas SAXS experiments were carried out with glycosylated LOX-PP. We generated theoretical 1D SAXS profile for each model using the Cα-Cα distances and the Gaussian smoothing as described in our recent work^31^. The models that best fitted the experimental 1D SAXS profile were models 1 and 2 with normalized χ^2^ values being 0.48 and 0.51 respectively. These values were 0.72, 0.59, and 0.88 for models 3, 4, and 5 respectively. These conformations were selected by the analysis, where the restraints were imposed on a single conformation and not on the ensemble of conformations simultaneously.

The secondary structures of all models were determined from the corresponding coordinate (.pdb) files. Models 1, 3 and 5 had the highest content of secondary structures (Table 1). 3_10_-helices and β-strands were found in the five models with a significant variation in 3_10_-helix content depending on the model. Topological diagrams showed that all models contained a few amount of secondary structures (4 to 9 β strands and 1 to 4 helices, Supplementary Fig. 5).

**Table 1:** Secondary structure content of LOX-PP models determined with PDBsum from their coordinate (.pdb) files.

LOX-PP models	β strand (%)	3_10_ helix (%)	Others (%)
1	9.6	11	79.4
2	6.8	6.2	87
3	9.6	12.3	78.1
4	11	3.4	85.6
5	13	11.6	75.3

### Heparin docking on LOX-PP and MD simulations

We have previously shown that LOX-PP interacts with 6-kDa HP with an affinity of 190 nM^26^ but the localization of the binding site of HP on LOX-PP is unknown. We have thus performed docking experiments with the 3D models of LOX-PP and a HP oligosaccharide in order to localize putative binding site(s) in LOX-PP sequence, and to determine if the oligosaccharide binding was able to induce LOX-PP folding. Poisson-Boltzmann surface area (PBSA) electrostatic potential calculations carried out for each model showed that arginine residues crucial for the positive electrostatic potential of LOX-PP were concentrated either at the N-terminus (models 1–3) or at the C-terminus (models 4–5). According to the computational analysis of all protein-GAG structures available in the Protein Data Bank (PDB, https://www.rcsb.org/) the regions with the positive electrostatic potential on the protein surface represent putative binding regions for negatively charged GAG molecules^32^. Therefore, we carried out docking simulations for each model of LOX-PP, sampling HP dp6 conformations in these regions. Contrasting results were obtained for LOX-PP/HP complexes depending on the model used for docking.

**Figure 7:**
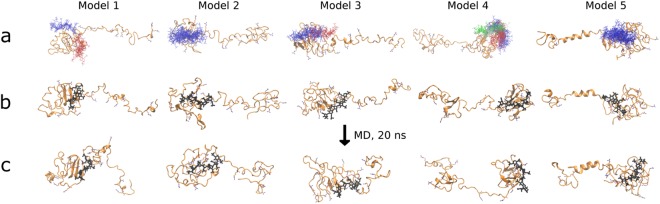
Docking and molecular dynamics of the heparin hexasaccharide/LOX-PP complex. (**a**) Clusters of docking solutions for heparin hexasaccharide binding to LOX-PP models obtained after the conversion from coarse-grained models to all atom representations (blue, red, green and cyan from the biggest cluster to the smallest one). The clusters were obtained from the top 50 docking solutions scored by AD3 with the DBSCAN algorithm (neighborhood search radius of 4 Å and minimal number of cluster members of 5). Structure of the LOX-PP/heparin hexasaccharide complex before (**b**) and after 20 ns all-atom MD simulations (**c**), corresponding to the lowest free energies for each model (Supplementary Table 3). Arginine residues: thin sticks, HP hexasaccharide: black thick sticks (bottom).

Representative clusters of docking solutions were either overlapping or localized in the same region of the propeptide (Fig. 7). A single, large, cluster, was obtained for models 2 and 5, two for models 1 and 3, and four for model 4. Both clusters were aligned but partially overlapped in model 3, whereas they were aligned end-to-end in model 1, forming an elongated binding site able to bind a longer chain of heparin. These data further indicate a high probability for HP multipose binding, which was previously computationally predicted and experimentally confirmed for other protein-GAG complexes^33–35^. HP predicted binding sites appeared thus to be mostly localized in globular regions, at the N-terminus for models 1–3 and at the C-terminus for models 4–5.

For each obtained cluster of docking solutions, we carried out all-atom MD simulations arbitrarily choosing three ligand poses from each cluster. In total, 30 different LOX-PP/HP complexes were analyzed. Again, our data suggest very heterogeneous nature of this interaction both for the free energy of binding and the radius of gyration changes upon GAG binding (Table 2). In most cases, the free energy of binding became more favorable during the simulation, suggesting that further contacts were established to tether LOX-PP/HP binding. The lowest energy values were obtained for models 1 and 2 complexed to HP dp6 (degree of polymerization), which suggests that they bound to the hexasaccharide with a higher affinity than the other models.

**Table 2:** MM-GBSA free energies of binding for LOX-PP/HP complexes after 10 and 20 ns of the simulation.

Model	Cluster	ΔG (kcal/mol) at 10 ns	ΔG (kcal/mol) at 20 ns	R_g_ (nm) at 10 ns	R_g_ (nm) at 20 ns
1	1	−110.4 ± 11.7	−128.6 ± 6.4	1.92 ± 0.04	1.78 ± 0.03
		−71.1 ± 6.5	−116.1 ± 7.1	2.62 ± 0.05	2.05 ± 0.04
		−80.3 ± 9.7	−133.0 ± 7.9	2.05 ± 0.03	1.96 ± 0.01
	2	−106.8 ± 10.6	−141.6 ± 11.5	2.44 ± 0.04	2.68 ± 0.06
		−138.8 ± 9.7	−162.9 ± 8.4	2.87 ± 0.07	2.27 ± 0.03
		−89.3 ± 8.8	−114.1 ± 8.3	3.21 ± 0.06	3.11 ± 0.05
2	1	−120.4 ± 10.6	−141.3 ± 7.2	2.25 ± 0.05	2.50 ± 0.08
		−98.2 ± 8.9	−109.1 ± 9.9	2.34 ± 0.05	2.16 ± 0.02
		−95.3 ± 8.2	−90.8 ± 6.6	2.11 ± 0.04	2.31 ± 0.04
3	1	−92.2 ± 7.6	−70.30 ± 7.5	2.22 ± 0.08	2.02 ± 0.04
		−55.7 ± 5.1	−72.6 ± 8.3	2.28 ± 0.03	1.92 ± 0.02
		−74.3 ± 7.8	−78.2 ± 7.1	1.91 ± 0.03	1.77 ± 0.03
	2	−74.3 ± 8.3	−85.4 ± 10.0	2.47 ± 0.05	2.39 ± 0.03
		−75.8 ± 7.4	−103.9 ± 8.1	2.09 ± 0.09	2.15 ± 0.05
		−73.3 ± 7.4	−81.5 ± 6.9	2.20 ± 0.03	2.25 ± 0.04
4	1	−43.6 ± 4.7	−70.9 ± 6.6	3.19 ± 0.03	3.26 ± 0.02
		−81.4 ± 5.6	−73.0 ± 5.8	2.37 ± 0.04	1.90 ± 0.03
		−78.4 ± 6.5	−75.0 ± 6.2	2.24 ± 0.06	2.07 ± 0.03
	2	−74.9 ± 5.6	−81.0 ± 6.2	2.96 ± 0.03	2.94 ± 0.04
		−54.4 ± 7.8	−56.0 ± 5.2	2.03 ± 0.06	2.24 ± 0.07
		−86.1 ± 9.5	−71.6 ± 6.6	3.05 ± 0.05	3.30 ± 1.00
	3	−71.3 ± 5.8	−75.8 ± 9.7	2.31 ± 0.03	2.28 ± 0.08
		−60.2 ± 6.2	−85.8 ± 6.9	2.16 ± 0.05	2.17 ± 0.04
		−77.4 ± 6.0	−91.3 ± 10.3	2.45 ± 0.05	2.79 ± 0.02
	4	−82.6 ± 7.7	−82.1 ± 6.7	3.21 ± 0.05	3.38 ± 0.08
		−45.4 ± 3.8	−66.9 ± 4.9	3.28 ± 0.03	3.30 ± 0.05
		−80.7 ± 5.6	−85.0 ± 6.8	3.28 ± 0.03	2.84 ± 0.03
5	1	−61.1 ± 7.4	−60.8 ± 5.7	2.13 ± 0.04	2.06 ± 0.05
		−50.9 ± 7.3	−71.0 ± 5.9	1.99 ± 0.06	1.83 ± 0.02
		−62.3 ± 5.7	−71.3 ± 5.9	2.25 ± 0.04	2.12 ± 0.06

Clusters were selected according to the procedure described in the Material and Methods section (n = 3).

We performed 20-ns all-atom MD simulations to determine if the binding of the heparin hexasaccharide induced a conformational change in LOX-PP (Supplementary Table 1). Although a trend towards a decrease in R_g_ values was observed, the difference did not reach statistical significance. Furthermore, the global decrease in R_g_ of LOX-PP during simulation may also result from spontaneous collapsing of LOX-PP due the force field used in this study as shown by MD simulations performed in the same conditions on all the models of LOX-PP alone (Supplementary Table 2). Consequently, the above data suggest that a 20-ns production simulation may be not long enough to allow statistically meaningful comparison of R_g_ values of LOX-PP upon heparin binding.

In addition, there were no significant differences (or obvious trends) in the secondary structure (α-helix and β-strand) of LOX-PP complexed with the HP hexasaccharide between 10-ns and 20-ns MD simulations in all the models (Supplementary Table 3), which is in agreement with CD spectra.

Molecular Mechanics Generalized Born and Surface Area (MM-GBSA) free energy per residue for LOX-PP/HP complexes was calculated from 30 independent MD simulations. Binding energy contributions ranged from 0.37 to 4.18 kcal/mol for acidic residues, from −0.37 to −15.24 kcal/mol for arginine residues, and from −0.16 to 0.05 kcal/mol for the only histidine residue (Supplementary Table 4). These values are within the range of those calculated using this approach for protein-GAG complexes of the PDB (average total energy: −74.3 ± 75.2 kcal/mol)^32^.

**Figure 8:**
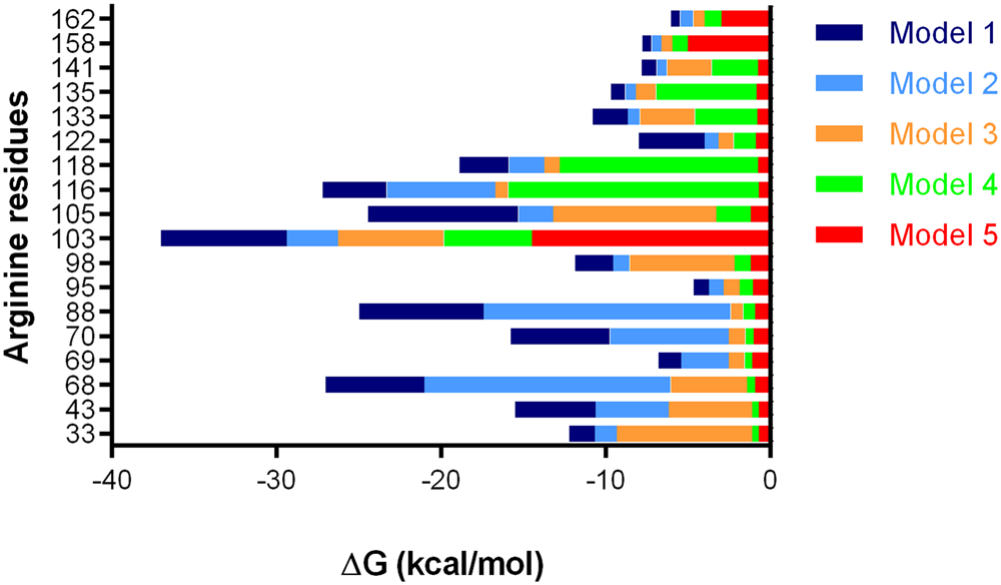
MM-GBSA free energy for all arginine residues of LOX-PP models bound to the heparin hexasaccharide.

The human propeptide does not contain lysine residues or any of the three consensus heparin-binding sites (XBBXBX, XBBBXXBX, and XBBBXXBBBXXBBX, where B is a basic residue and X is a hydropathic residue^36^). These data showed the crucial role of several arginine residues in the hexasaccharide binding, which were the only basic residues of LOX-PP. Two binding sites were determined, a major one encompassing the R103-R118 sequence for models 1–4 (R103 being the most frequent contributor), and another one encompassing the R68-R88 sequence mostly in models 1 and 2 (Fig. 8 and Supplementary Fig. 6). One out of the four regions of LOX-PP predicted by ANCHOR to fold upon partner binding (38–65, 74–114, 118–139, 149–168) encompassed the major part of both predicted binding sites. The regions of LOX-PP predicted to fold by MoRFchibi SYSTEM (37–61, 63–70, 84–132, and 153–158) also overlapped with the predicted binding sites.

### New binding partners: the interaction network of human LOX-PP

**Figure 9:**
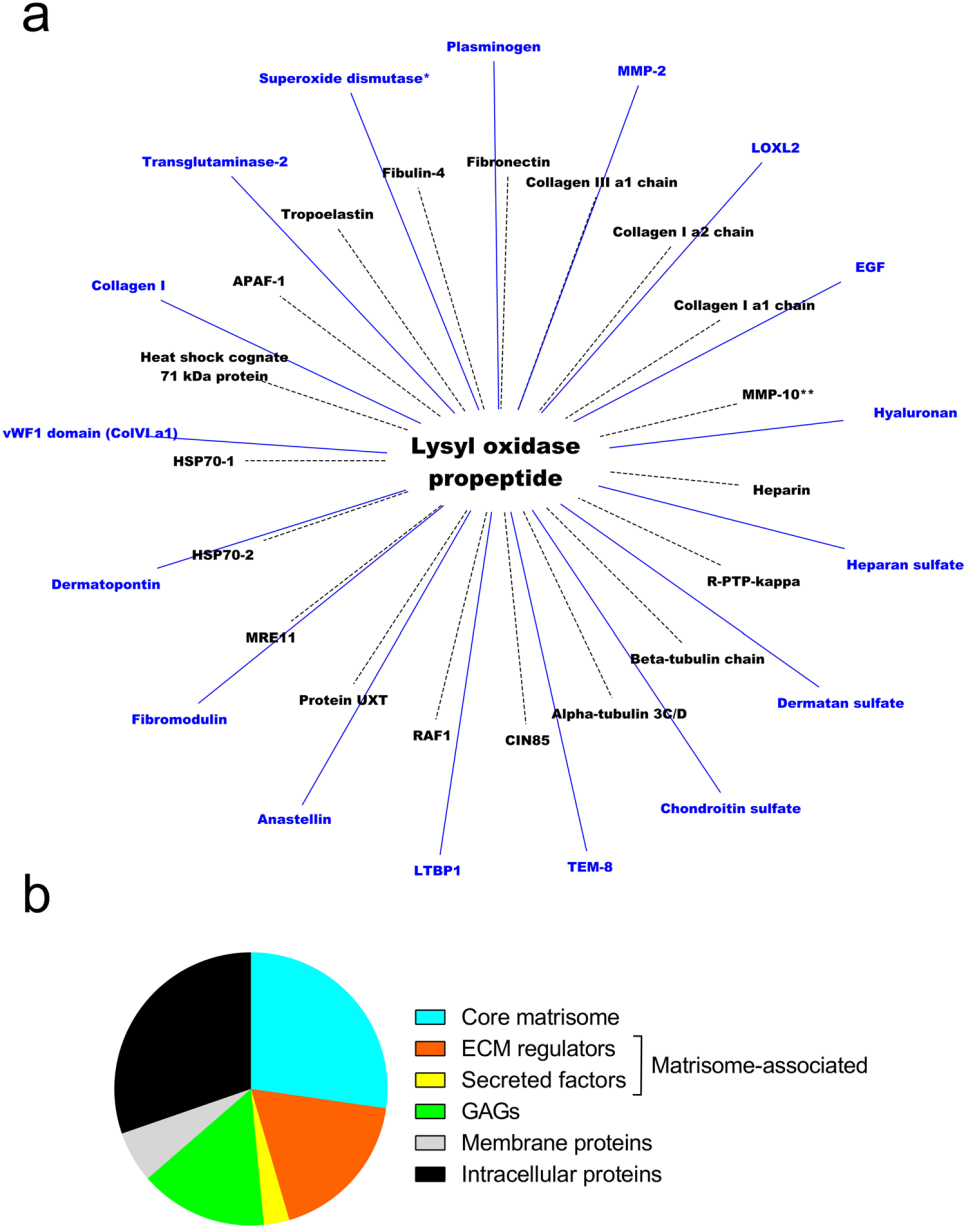
Interaction network of human LOX-PP. (**a**) Protein-protein and protein-GAG interaction network of LOX-PP. Previously identified partners are linked to LOX-PP by dashed edges, whereas those identified in this study are indicated by solid edges. *Protein from *Leishmania major*. **A direct interaction between MMP-10 and LOX-PP has not been demonstrated but the sequence cleaved by MMP-10 has been located in the propeptide^25^. APAF-1: apoptotic protease-activating factor 1, CIN85: Cbl-interacting protein of 85 kDa (SH3 domain-containing kinase-binding protein 1), MRE11: double-strand break repair protein MRE11, Protein UXT: Ubiquitously expressed transcript protein, R-PTP-kappa: Receptor-type tyrosine-protein phosphatase kappa, RAF1: proto-oncogene serine/threonine-protein kinase, TEM-8: tumor endothelial marker-8). (**b**) Pie chart representation of LOX-PP partners based on Naba’s ECM classification^84^. Collagen I trimer, but not its isolated α chains, was included in the chart.

To determine if LOX-PP was able to bind to other partners we first investigated if it interacted with other GAGs and to ECM proteins. Indeed, most partners of LOX-PP identified at the time we started this work were intracellular. The partners of LOX-PP reported in the literature are listed in Supplementary Table 5. Potential partners of LOX-PP were screened by Surface Plasmon Resonance imaging (SPRi), SPR, and Bio-Layer Interferometry (BLI) (Supplementary Tables 6 and 7). We have identified 17 new partners of LOX-PP including four GAGs (chondroitin sulfate, dermatan sulfate, heparan sulfate, hyaluronan), collagen I, cross-linking and proteolytic enzymes (lysyl oxidase-like 2, transglutaminase-2, and MMP-2), one proteoglycan (fibromodulin), one matricryptin (anastellin), and the ectodomain of one membrane protein (Tumor Endothelial Marker-8 also known as anthrax receptor-1^37^). A single representative evidence of each interaction is displayed in Supplementary Figs 7 and 8. The interaction network of LOX-PP integrating partners curated from the literature and available in MatrixDB database^38^ (http://matrixdb.univ-lyon1.fr/) is displayed in Fig. 9a. All the interactions identified in this study will be publicly and freely available in MatrixDB database. Most LOX-PP partners belong to the extracellular matrix (42.5%) or are associated with it (21.2%), whereas 30.3% of them are intracellular and 6.1% are membrane proteins (Fig. 9b). Moreover, LOX-PP bound with very high affinity to tropoelastin and to plasminogen, and with high affinity to anastellin, a fragment of fibronectin (Table 3, Supplementary Fig. 8b).

**Table 3:** Kinetic parameters, equilibrium dissociation constant (K_D_), and binding models of LOX-PP interactions with its partners calculated from Bio-Layer Interferometry (BLI) and Surface Plasmon Resonance (SPR) binding assays.

Immobilized partner	Analyte	ka	kd (s^−1^)	K_D_ (nM)	Model
Anastellin	LOX-PP	2.93 × 10^3^ M^−1^s^−1^	4.45 × 10^−4^	152	1:1 (BLI)
Tropoelastin	LOX-PP	3.31 × 10^2^ M^−1^s^−1^ 3 × 10^−3^ s^−1^	4.3 × 10^−3^ 3.1 × 10^−7^	1.39	2-state (SPR)
LOX-PP	Plasminogen	4.17 ± 0.74 × 10^4^ M^−1^s^−1^ 2.14 ± 0.05 × 10^−3^ s^−1^	5.15 ± 0.21 × 10^−3^ 3.06 ± 0.16 × 10^−4^	15.8 ± 1.75	2-state (SPR) n = 2

## Discussion

Human LOX-PP is predicted to contain more than 80% of disordered residues, which is similar to the value reported for the rat LOX-PP (67% using another predictor^27^). The presence of intrinsic disorder has been confirmed experimentally by CD and SAXS experiments. However, LOX-PP is partially structured as shown by deconvolution of CD spectra, which demonstrated the presence of a low amount of α-helix and β-strands. The 3D models of the propeptide are consistent with the fact that the propeptide is not entirely disordered. Indeed, all models (except for model 5, which has the lowest probability score) contain secondary structures mostly located at the N- and C-termini and a disordered central part. The most probable models (1–3) contain a globule-like domain at the N-terminus, which is consistent with the fact that the amino acid sequence 44–58 is predicted to be structured. This sequence is located in a globular region in models 1–4, and in the N-terminal helical part in model 5. The most probable model (model 1) comprises 11% of 3_10_-helix and 9.6% of β-strands compared to 3.4% of α-helix and 20.4% of β-strands determined by CD experiments. N-glycosylated^39^ (N81, N97, and N144) and putative O-glycosylated^40^ residues (T89, T113, T120, S130, T131, S132) are located in the disordered region of human LOX-PP, as expected for glycosylation. Indeed, the post-translational modification glycosylation has been shown to be strongly correlated with predicted disorder^41^. The combination of DLS and SAXS data shows that LOX-PP is an elongated, flexible, protein with a D_max_ of 11.7 nm and a R_g_ of 3.7 nm, which is consistent with the R_g_ value calculated for model 1 (2.93 nm). The flexibility of LOX-PP is further emphasized by the predicted existence in solution of several conformers with a 2-fold variation in the D_max_ value, and the normalized Kratky plot.

LOX-PP can adopt an α-helical structure in presence of trifluoroethanol as reported for the rat propeptide^27^. This prompted us to determine if the propeptide was able to fold upon HP binding. No significant change was observed in CD spectra, and hence in the secondary structure of LOX-PP in presence of full-length HP or of a hexasaccharide. Docking experiments with a hexasaccharide and MD simulations with the LOX-PP models generated by SAXS-data-restrained coarse-grained simulations also failed to show any significant changes in LOX-PP secondary structure and conformation.

The models 1 and 2, which are the most probable ones according to coarse-grained simulations, bind stronger to the HP hexasaccharide. The top 10 arginine residues involved in HP binding (R116, R103, R105, R118, R68, R88, R122, R133, R135, and R43 in decreasing order of their contribution) are located in disordered regions of the propeptide, which are predicted to fold upon binding both by ANCHOR and MoRFchibi SYSTEM. The formation of α-helix observed in the presence of trifluoroethanol could thus occur in the HP-binding region located between R103 and R122.

The 3D models of LOX-PP bound to the hexasaccharide show that the sequence 26–100, which is involved in the interaction of LOX-PP with c-Raf and HSP70^18^, is globular and contains two accessory arginine residues (R68, R88), which may contribute to heparin binding. The cleavage sites of LOX-PP by MMP-2 (Asn150-Leu151 in rat, and Asn156-Leu157 in human) and BMP-1^24^ (Gly168-Asp169) and the arginine residue R158, crucial for the adipogenic effect of LOX-PP, are not located in the HP-binding region. This residue plays an important role in LOX-PP functions because its mutation in glutamine inhibits the adipogenic effect of LOX-PP^14^ and impairs the ability of LOX-PP to inhibit the invasive phenotype and tumor formation of breast cancer cells^42^. In contrast to the above residues, the cleavage site of MMP-10 (Ser56-Leu57)^25^, and P111 and R116, which are required for LOX-PP binding to CIN85^19^, are in the HP-binding region. CIN85 being a cytoplasmic protein it is unlikely that HP could interfere with its binding to LOX-PP *in vivo*. Furthermore, LOX-PP is predicted to bind to the SUMO protein, which contributes to DNA lesion reparation in mammalian cells^43^, *via* the PILL sequence (residues 90–93) with a score of 0.795 using JASSA^44^ (http://www.jassa.fr/index.php?m=jassa). This potential interaction may play a role in LOX-PP binding to nuclear proteins.

We report here that LOX-PP interacts with chondroitin, dermatan and heparan sulfate and with hyaluronan. It may thus interact with cell surface proteoglycans such as syndecans and glypicans, and contribute to cell-matrix interactions. In addition, LOX-PP has intracellular and extracellular protein partners and appears to be a moonlighting protein fulfilling different roles inside and outside the cells. LOX-PP binds to ECM proteins, namely collagen I, the α1 chain of collagen VI *via* its von Willebrand Factor (vWF) domain 1, the small leucine-rich proteoglycan fibromodulin, fibronectin and its anastellin fragment, and Latent-Transforming growth factor beta Binding Protein-1 (LTBP-1) *via* its EGF domains. LOX-PP likely participates in collagen fibrillogenesis in association with fibromodulin, in fibronectin supramolecular assembly and in elastic fiber formation. Several LOX-PP partners such as fibrillar collagen I^45^, elastin^10^, fibronectin^23^, dermatopontin^46,47^, and fibromodulin^48^ bind to mature LOX, suggesting that both mature LOX and the propeptide are involved in ECM assembly and organization. LOX-PP is sufficient to promote the binding of prolysyl oxidase to MMP-2, which has been previously reported^24^, and it interacts with the cross-linking enzymes lysyl oxidase-like 2 (LOXL-2), and transglutaminase-2. LOX-PP contains 14 glutamine residues, and may be a glutaminyl substrate of transglutaminase-2. The interaction of the propeptide with the pro-angiogenic growth factor EGF and with TEM-8, which is overexpressed at the surface of activated endothelial cells, suggests its possible involvement in angiogenesis, and possible interference with EGF signaling pathway. Given that TEM-8 is one of the receptors of the anthrax toxin and that it binds to live *Leishmania* parasites^49^, LOX-PP might also contribute to the interactions between the host ECM and pathogens. Proteins containing EGF, vWF and fibronectin domains, found in LOX-PP partners, are potential partners of the propeptide, and are currently investigated to increase the coverage of the propeptide interaction network.

## Material and Methods

### Expression of recombinant LOX-PP

LOX-PP (UniProtKB identifier of LOX: P28300, UniProtKB pro feature of LOX-PP PRO_0000018520 encompassing residues 22–168 with the substitution P24L) was expressed with a C-terminal FLAG tag in human embryonic kidney 293 Epstein-Barr Nuclear Antigen cells (HEK293-EBNA) using the pCEP-Pu-BM40 vector^50^. Cells were selected with puromycin (Sigma-Aldrich, P9620, 0.5 µg/ml) and expanded in Dulbecco’s Modified Eagle’s medium (DMEM) high glucose medium (Sigma-Aldrich, D5796) containing 50 µg/ml of gentamicin (Sigma-Aldrich, G1272). Culture media were harvested every 48 h for fourteen days. cOmplete^TM^ ethylenediamine tetraacetic acid (EDTA)-free Protease Inhibitor (Roche) and 0.1 mM phenylmethylsulfonyl fluoride were added to the medium which was then centrifuged at 14 000 × *g* for 25 min at 4 °C and stored at −80 °C until purification. The protein was purified by affinity chromatography on an anti-FLAG resin (Sigma-Aldrich, A2220) in 10 mM Hepes, 150 mM NaCl pH 7.4 (Hepes buffered saline, HBS), eluted by the FLAG peptide (200 µg/ml in HBS), and concentrated up to 6.6 mg/ml (219 µM) by centrifugation using an Amicon Ultra-0,5 device (Merck Millipore, UFC5010, 10 kDa cut-off). The purification process was followed by SDS-PAGE and Western blot with a primary anti-FLAG antibody (F3165, Sigma-Aldrich^51^) and a secondary antibody conjugated to peroxidase (Bio-Rad, 172–1011). The gels were stained with EZBlue™ Gel Staining Reagent and were scanned with the Canon LiDE 210 scanner. Immunocomplexes were detected in Western blot with SuperSignal West Pico Chemiluminescence Substrate (34080, Thermo Scientific) and visualized using the Fusion FX camera (Vilber-Lourmat) for three minutes with default settings. Pre-stained molecular weight markers were also visualized with the Fusion FX camera (Vilber-Lourmat), and superimposed on the membrane imaged by chemiluminescence with the Fusion Capt Advance software. The identity of the purified propeptide was controlled by Liquid Chromatography/Mass spectrometry-Mass spectrometry (UMS 3444/US8, Lyon, France).

### Prediction of intrinsic disorder in LOX-PP

Intrinsically disordered regions in the full-length sequence of LOX-PP were predicted with the metapredictor metaPrDOS combining DISOPRED2, DISPROT, IUPred, and PrDOS predictors^52^ (http://prdos.hgc.jp/cgi-bin/meta/top.cgi). Molecular recognition features, short disordered regions able to undergo disorder-to-order transition upon binding to partners, were predicted in LOX-PP with ANCHOR^53^ (http://anchor.enzim.hu/) and MoRFchibi SYSTEM^54^ (http://www.chibi.ubc.ca/faculty/joerg-gsponer/gsponer-lab/software/morf_chibi/).

### Circular dichroism

The propeptide of lysyl oxidase was dialyzed against 10 mM potassium phosphate pH 7.4 (2.0 µM, 60 µg/ml) and analyzed at 20 °C in a Chirascan system (Applied Photophysics, UMS 3444/US8, Lyon, France). Spectra of LOX-PP and buffer were also recorded in presence of increasing concentrations (20, 40, 60, and 80%, v/v) of 2,2,2-trifluoroethanol (TFE, Roth, CP29). A series of experiments was performed in the same experimental conditions in the presence of full-length HP (Sigma-Aldrich, H3393, 17 kDa) and HP hexasaccharide (dp6, Iduron, HO06, 1.8 kDa). LOX-PP (2 µM) and HP (2 µM) or a HP hexasaccharide (2 µM) were mixed at a molar ratio of 1:1 immediately before the experiment. Further spectra were collected after incubating the mixture at room temperature for one hour. For all experiments five consecutive spectra were recorded in a 1-mm pathlength cell for both LOX-PP and buffer, and averaged. The buffer spectra were subtracted from LOX-PP spectra. The resulting spectra were then smoothed and deconvoluted with the CONTIN algorithm^55,56^ and the data set 7, which contains five denatured proteins on DichroWeb^57^ (http://dichroweb.cryst.bbk.ac.uk/html/home.shtml).

### Dynamic Light Scattering (DLS)

DLS measurements were performed in a Zetasizer Nano ZS (Malvern, UMR 5223, Villeurbanne, France) using a disposable plastic cuvette (Eppendorf, 0030106300). Four series of fifteen spectra of LOX-PP diluted to 1.1 mg/ml (35 µM) in HBS were acquired at a 173° angle, at 633 nm, at 20 °C. Data were analyzed with the Malvern software.

### Size-exclusion chromatography - Multi-angle light scattering (SEC-MALS)

LOX-PP diluted at 75 µM (2.3 mg/ml) in HBS was centrifuged 5 min at 14000 × *g* and 25 µl of the supernatant were injected on a Superdex 200 Increase 5/150 GL column (GE Healthcare) at a flow rate of 0.2 ml/min at 25 °C. The elution was followed by UV absorbance at 280 nm, multi-angle light scattering at 690 nm (MALS, miniDAWN-TREOS detector, Wyatt Technology Corp.) and refractometry (RI, Optilab T-rex system, Wyatt Technology). ASTRA software (Wyatt Technology) was used to calculate molecular weights.

### Small-Angle X-ray Scattering (SAXS)

SAXS experiments (MX-1841, MX-1920) were performed on the BM29 BioSAXS beamline at the European Synchrotron Radiation Facility^58,59^ (ESRF, Grenoble, France). Before analysis, LOX-PP solution was centrifuged at 14 000 × *g* for 12 min to eliminate potential aggregates. LOX-PP was injected at two different concentrations (37.4 and 18.7 µM, 1.12 and 0.56 mg/ml respectively in HBS) in the 1.8 mm-diameter quartz capillary using the automatic robotic sample changer. Ten frames per concentration were collected at 20 °C at an exposure time of 0.5 s per frame and a flow of 5 µl/s with a 0.9919 Å wavelength on a Pilatus 1 M detector (beam center 888 × 91 pixel, 172 µm/pixel) at 2.867 m from the sample to cover a range of momentum transfer between 0.031 and 4.94 nm^−1^. Data quality was assessed with the autoprocessing framework EDNA^60^ before analysis. Buffer spectra were subtracted from LOX-PP spectra and data were extrapolated to infinite concentration using ATSAS suite^61^. Useful data range was defined by SHANUM^62^ and PRIMUS^63^ was used to calculate the radius of gyration of LOX-PP through Guinier’s approach with a sR_g_ limit <1.06. Normalized Kratky plot was generated with scÅtter^64^ to evaluate the flexibility of LOX-PP. The theoretical R_g_ was calculated with Flory’s equation, which associates the radius of gyration with the protein length (R_g_ = R_0_N^ν^, N being the number of residues of the protein, R_0_ being 2.54 ± 0.01 and ν 0.522 ± 0.01 for intrinsically disordered proteins^65^).

Ensemble modelling was performed with EOM (Ensemble Optimization Method 2.1), which is adapted for ensemble modelling of flexible macromolecules^30^. EOM was run to analyze the behavior of LOX-PP compared to a gaussian pool of different conformations generated from the propeptide sequence. The following parameters were used: core symmetry p1, native-like structure because LOX-PP is a disordered protein, number of harmonics: 50, number of points: 100. 20 000 independent models were generated. The genetic algorithm was run 100 times to compare the averaged theoretical scattering intensity from independent ensembles of conformations against the experimental scattering data.

### Surface Plasmon Resonance imaging (SPRi) binding assays

SPRi experiments were performed on a Biacore Flexchip system (GE Healthcare, UMS 3444/US8, Lyon, France) at 25 °C as previously described^26,66,67^. Briefly, 93 unique proteins, protein domains and GAGs were spotted in triplicate on a bare gold chip (Gold Affinity chip, GE Healthcare) at concentrations ranging from 0.06 to 1 mg/ml with a non-contact spotter (sciFlexarrayer S3, Scienion) (Supplementary Table 6). The chips were then blocked for 5 × 5 min (Flexchip blocking buffer, GE Healthcare) and equilibrated in phosphate-buffered saline containing 0.05% Tween 20 (Sigma-Aldrich, P2287) at a flow rate of 300 µl/min for 90 min. Purified LOX-PP was injected at 2 µM in the same buffer and recirculated for 20 min over the chips. The dissociation of the complexes was followed in running buffer for 40 min. Kinetics and affinity of some interactions identified by SPRi were calculated either by SPR or Bio-Layer Interferometry (BLI).

### Surface Plasmon Resonance binding assays

SPR binding assays were performed in Biacore T100 or T200 systems (GE Healthcare, UMS 3444/US8, Lyon, France) at 25 °C in HBS pH 7.4 containing 0.05% P20 (HBS-P20, GE Healthcare, BR100054). Proteins were covalently immobilized onto CM5 sensor chips (series S, GE Healthcare) *via* their amino groups as previously described^68^. Proteins (Supplementary Table 6) were injected over the activated sensor chips for 2 to 10 min at concentrations ranging from 50 to 500 µg/ml. Residual activated groups were blocked by injecting 1 M ethanolamine pH 8.5 for 7 min. A control flow cell was prepared by omitting the protein during the immobilization process. Analytes (Supplementary Table 6) were injected at 30 µl/min for 3 min on immobilized ligands and on the control flow cell to assess non-specific binding to the sensor chip surface. Regeneration was carried out by injecting 1 M NaCl, 1.5 M NaCl or 1.5 M NaCl containing 1.5 M guanidinium chloride for 15 to 30 s. Single-cycle kinetics were performed to determine affinity (K_D_) and kinetics parameters (k_a_, k_d_), which were calculated with the BiaEval software.

### Bio-Layer Interferometry (BLI) binding assays

Interactions experiments were performed on an Octet RED96 system (Pall, ForteBIO) using amine reactive second generation (AR2G) biosensors. Sensors were hydrated in water for 10 min, then carboxylic groups were activated for 10 min with 0.1 M N-hydroxysulfosuccinimide and 0.2 M 1-ethyl-3-(3-dimethylaminopropyl)carbodiimide hydrochloride. Anastellin (Sigma-Aldrich, F3542, 100 µg/ml) was covalently coupled to the sensor surface *via* its primary amine groups in 10 mM sodium maleate pH 6.2 for 10 min. Residual activated groups were blocked with 1 M ethanolamine pH 8.5 for to 10 min. The sensors were equilibrated in HBS-P20, and then dipped into LOX-PP solutions for 3 min. Sensors were regenerated by dipping in 1 M NaCl, 1.5 M NaCl or 1.5 M NaCl containing 1.5 M guanidinium chloride. Data were analyzed with Evaluation 9.0 software.

### Building and analyzing the interaction network of LOX-PP

The interaction network of LOX-PP was built by integrating the new partners identified in this study by SPRi, SPR and BLI, and those curated from the literature available in the interaction database MatrixDB^38^ (http://matrixdb.univ-lyon1.fr/) we have created. The network was visualized using Cytoscape^69^ (http://www.cytoscape.org/).

### Coarse-grained simulations of the lysyl oxidase propeptide

3D models of LOX-PP were generated using the UNRES coarse-grained model of proteins^70,71^ with the recently added function of including the distance distribution from SAXS as restraints^31^. The full protocol is detailed in Supplementary material. The secondary structure of each model was determined from the coordinate (.pdb) files of the models with PDBsum^72^ (http://www.ebi.ac.uk/pdbsum). Topological diagrams were generated with PROMOTIF software, which is based on the Dictionary of Secondary Structure of Proteins (DSSP)^73^. The full-length sequence of LOX-PP (UniProtKB pro feature PRO_0000018520) encompassing residues 22–168 was used for coarse-grained simulations.

### Molecular docking

The five models of LOX-PP obtained by UNRES coarse-grained MD simulations were used for docking of a HP hexasaccharide (GlcNS(6S)-IdoUA(2S))_3_ with Autodock 3^74^ (AD3), which yields the best performance among other docking programs for GAG ligands^32^. Charges of LOX-PP atoms were assigned by AD3, and charges of HP dp6 were taken from the corresponding AMBER residue libraries^75^ compatible with the GLYCAM06 force field^76^. IdoUA(2S) ring in ^1^C_4_ conformation was used. The following parameters for AD3 local docking runs were applied: a grid spacing of 0.4 Å step with 126 × 126 × 126 grid points as dimensions of the box, which contains the putative HP binding region according to PBSA electrostatic potential calculations in AMBER. Electrostatic potential isosurfaces were calculated using the PBSA program from AmberTools with a grid spacing of 1 Å. Dielectric constants for the solvent and the solute were 80 and 1 respectively, and the surface tension parameter for nonpolar solvation calculation was 0.005^77^. Lamarckian genetic algorithm (initial population size of 300, 10^5^ generations, 9995 × 10^5^ energy evaluations), 10^3^ independent runs were used for molecular docking. All covalent bonds with the exception of N-S and O-S for N- and O-sulfate groups, were kept flexible, corresponding to 30 torsional angle degrees of freedom in total. 50 top docking solutions scored by AD3 out of 10^3^ obtained docked structures for each docking experiment were clustered by the DBSCAN algorithm^78^ with a neighborhood search radius of 4 Å and minimal number of cluster members of 5. The metric distance between two structures was defined as the root mean square of atomic distances (RMSD) for the nearest atoms of the same type in order to take into account GAG periodic nature^79^. Three arbitrarily chosen poses from each cluster were used for MD-based refinement and analysis. Prior to MD simulations, the conformations of GAG glycosidic linkages were checked to avoid distorting starting geometries that could be potentially observable in the structures produced by AD3^80^.

### Molecular dynamics

MD simulations of LOX-PP/HP complexes obtained by molecular docking were carried out in AMBER16^77^ (http://ambermd.org/). We used periodic boundary conditions with a truncated octahedron TIP3P water box with at least 4 Å distance from the solute to the periodic box border, which corresponded to 2–3 × 10^5^ water molecules in the periodic box, and counterions. ff99SB force field parameters for protein and the GLYCAM06^76^ for GAGs were used. Two energy-minimization steps were carried out: 0.5 × 10^3^ steepest descent cycles and 10^3^ conjugate gradient cycles with harmonic force restraints on solute atoms, 3 × 10^3^ steepest descent cycles and 3 × 10^3^ conjugate gradient cycles without restraints. Then, the system was heated up to 300 K for 10 ps, equilibrated for 50 ps at 300 K and 10^6^ Pa in isothermal isobaric ensemble (NPT). Finally, a 20 ns of productive MD run was carried out in NTP ensemble. The SHAKE algorithm, 2 fs time integration, 8 Å cutoff for non-bonded interactions and the Particle Mesh Ewald method were used. All MD simulations of LOX-PP/HP complexes were repeated three times. Analysis of the trajectories was done using the cpptraj module of AMBER Tools 17^77^ (http://ambermd.org/). The radius of gyration was calculated for all protein atoms using radgyr command with default parameters in cpptraj. VMD^81^ was used for visualization of trajectories and the figures. Energetic post-processing of the trajectories and per residue energy decomposition were done using MM-GBSA with igb = 2 for protein-GAG complexes. Entropic contribution to binding was not considered^82,83^. Therefore, the calculated free energies should be understood as enthalpies which also implicitly partially account for the entropic component of the solvent in the implicit model.

## Electronic supplementary material

Supplementary material

## References

[1] Mäki JM (2009). Lysyl oxidases in mammalian development and certain pathological conditions. Histol. Histopathol..

[2] Baker A-M (2013). Lysyl oxidase plays a critical role in endothelial cell stimulation to drive tumor angiogenesis. Cancer Res..

[3] Cox TR, Gartland A, Erler JT (2016). Lysyl Oxidase, a Targetable Secreted Molecule Involved in Cancer Metastasis. Cancer Res..

[4] Trackman PC (2016). Enzymatic and non-enzymatic functions of the lysyl oxidase family in bone. Matrix Biol..

[5] Johnston KA, Lopez KM (2018). Lysyl oxidase in cancer inhibition and metastasis. Cancer Lett..

[6] Trackman, P. C. Functional importance of lysyl oxidase family propeptide regions. *J*. *Cell*. *Commun*. *Signal*. 10.1007/s12079-017-0424-4 (2018). 10.1007/s12079-017-0424-4 PMC5842187 29086201

[7] Ricard-Blum S, Vallet SD (2016). Proteases decode the extracellular matrix cryptome. Biochimie.

[8] Ricard-Blum, S. & Vallet, S. D. Fragments generated upon extracellular matrix remodeling: Biological regulators and potential drugs. *Matrix Biol*. 10.1016/j.matbio.2017.11.005 (2017). 10.1016/j.matbio.2017.11.005 29133183

[9] Grimsby JL, Lucero HA, Trackman PC, Ravid K, Kagan HM (2010). Role of lysyl oxidase propeptide in secretion and enzyme activity. J. Cell. Biochem..

[10] Thomassin L (2005). The Pro-regions of lysyl oxidase and lysyl oxidase-like 1 are required for deposition onto elastic fibers. J. Biol. Chem..

[11] Palamakumbura AH (2004). The propeptide domain of lysyl oxidase induces phenotypic reversion of ras-transformed cells. J. Biol. Chem..

[12] Hurtado PA (2008). Lysyl oxidase propeptide inhibits smooth muscle cell signaling and proliferation. Biochem. Biophys. Res. Commun..

[13] Vora SR (2010). Lysyl oxidase propeptide inhibits FGF-2-induced signaling and proliferation of osteoblasts. J. Biol. Chem..

[14] Griner JD, Rogers CJ, Zhu M-J, Du M (2017). Lysyl oxidase propeptide promotes adipogenesis through inhibition of FGF-2 signaling. Adipocyte.

[15] Bais MV, Ozdener GB, Sonenshein GE, Trackman PC (2015). Effects of tumor-suppressor lysyl oxidase propeptide on prostate cancer xenograft growth and its direct interactions with DNA repair pathways. Oncogene.

[16] Li J (2010). Nna1 mediates Purkinje cell dendritic development *via* lysyl oxidase propeptide and NF-κB signaling. Neuron.

[17] Guo Y, Pischon N, Palamakumbura AH, Trackman PC (2007). Intracellular distribution of the lysyl oxidase propeptide in osteoblastic cells. Am. J. Physiol. Cell Physiol..

[18] Sato S (2011). The Ras signaling inhibitor LOX-PP interacts with Hsp70 and c-Raf to reduce Erk activation and transformed phenotype of breast cancer cells. Mol. Cell. Biol..

[19] Sato S (2013). Inhibition of CIN85-mediated invasion by a novel SH3 domain binding motif in the lysyl oxidase propeptide. PLoS ONE.

[20] Sánchez-Morgan N, Kirsch KH, Trackman PC, Sonenshein GE (2017). UXT Is a LOX-PP Interacting Protein That Modulates Estrogen Receptor Alpha Activity in Breast Cancer Cells. J. Cell. Biochem..

[21] Sánchez-Morgan N, Kirsch KH, Trackman PC, Sonenshein GE (2011). The lysyl oxidase propeptide interacts with the receptor-type protein tyrosine phosphatase kappa and inhibits β-catenin transcriptional activity in lung cancer cells. Mol. Cell. Biol..

[22] Ozdener GB, Bais MV, Trackman PC (2016). Determination of cell uptake pathways for tumor inhibitor lysyl oxidase propeptide. Mol. Oncol..

[23] Fogelgren B (2005). Cellular fibronectin binds to lysyl oxidase with high affinity and is critical for its proteolytic activation. J. Biol. Chem..

[24] Panchenko MV, Stetler-Stevenson WG, Trubetskoy OV, Gacheru SN, Kagan HM (1996). Metalloproteinase activity secreted by fibrogenic cells in the processing of prolysyl oxidase. Potential role of procollagen C-proteinase. J. Biol. Chem..

[25] Schlage P (2014). Time-resolved analysis of the matrix metalloproteinase 10 substrate degradome. Mol. Cell Proteomics.

[26] Vallet, S. D. *et al*. Chapter 11: Strategies for Building Protein–Glycosaminoglycan Interaction Networks Combining SPRi, SPR, and BLI. In *Handbook of Surface Plasmon Resonance*, 398–414, 10.1039/9781788010283-00398 (2017).

[27] Vora SR (2010). Characterization of recombinant lysyl oxidase propeptide. Biochemistry.

[28] Fischer H (2010). Determination of the molecular weight of proteins in solution from a single small-angle X-ray scattering measurement on a relative scale. J. Appl. Cryst..

[29] Burchard, W. Static and dynamic light scattering from branched polymers and biopolymers. in *Laser light scattering in biochemistry*. 3–22 (Harding, S. E., Settele, D. B. & Bloomfield, V. A. editors, 1992).

[30] Tria G, Mertens HDT, Kachala M, Svergun DI (2015). Advanced ensemble modelling of flexible macromolecules using X-ray solution scattering. IUCrJ.

[31] Karczyńska AS (2018). Prediction of protein structure with the coarse-grained UNRES force field assisted by small X-ray scattering data and knowledge-based information. Proteins.

[32] Samsonov SA, Pisabarro MT (2016). Computational analysis of interactions in structurally available protein-glycosaminoglycan complexes. Glycobiology.

[33] Atkovska K, Samsonov SA, Paszkowski-Rogacz M, Pisabarro MT (2014). Multipose Binding in Molecular Docking. Int. J. Mol. Sci..

[34] Joseph PRB, Mosier PD, Desai UR, Rajarathnam K (2015). Solution NMR characterization of chemokine CXCL8/IL-8 monomer and dimer binding to glycosaminoglycans: structural plasticity mediates differential binding interactions. Biochem. J..

[35] Rother S (2016). Structural and functional insights into the interaction of sulfated glycosaminoglycans with tissue inhibitor of metalloproteinase-3 - A possible regulatory role on extracellular matrix homeostasis. Acta Biomater..

[36] Capila I, Linhardt RJ (2002). Heparin-protein interactions. Angew. Chem. Int. Ed. Engl..

[37] Cryan LM, Rogers MS (2011). Targeting the anthrax receptors, TEM-8 and CMG-2, for anti-angiogenic therapy. Front. Biosci..

[38] Launay G, Salza R, Multedo D, Thierry-Mieg N, Ricard-Blum S (2015). MatrixDB, the extracellular matrix interaction database: updated content, a new navigator and expanded functionalities. Nucleic Acids Res..

[39] Trackman PC, Bedell-Hogan D, Tang J, Kagan HM (1992). Post-translational glycosylation and proteolytic processing of a lysyl oxidase precursor. J. Biol. Chem..

[40] Steentoft C (2011). Mining the O-glycoproteome using zinc-finger nuclease-glycoengineered SimpleCell lines. Nat. Methods.

[41] Xie H (2007). Functional anthology of intrinsic disorder. 3. Ligands, post-translational modifications, and diseases associated with intrinsically disordered proteins. J. Proteome Res..

[42] Min C (2009). A loss-of-function polymorphism in the propeptide domain of the LOX gene and breast cancer. Cancer Res..

[43] Garvin, A. J. & Morris, J. R. SUMO, a small, but powerful, regulator of double-strand break repair. *Philos*. *Trans*. *R*. *Soc*. *Lond*. *B*. *Biol*. *Sci*. **372** (2017). 10.1098/rstb.2016.0281 PMC5577459 28847818

[44] Beauclair G, Bridier-Nahmias A, Zagury J-F, Saïb A, Zamborlini A (2015). JASSA: a comprehensive tool for prediction of SUMOylation sites and SIMs. Bioinformatics.

[45] Siegel R (1974). Biosynthesis of collagen crosslinks: increased activity of purified lysyl oxidase with reconstituted collagen fibrils. PNAS.

[46] Cronshaw AD (1993). TRAMP (tyrosine rich acidic matrix protein), a protein that co-purifies with lysyl oxidase from porcine skin. Identification of TRAMP as the dermatan sulphate proteoglycan-associated 22K extracellular matrix protein. Matrix.

[47] Forbes EG, Cronshaw AD, MacBeath JR, Hulmes DJ (1994). Tyrosine-rich acidic matrix protein (TRAMP) is a tyrosine-sulphated and widely distributed protein of the extracellular matrix. FEBS Lett..

[48] Kalamajski S, Bihan D, Bonna A, Rubin K, Farndale RW (2016). Fibromodulin Interacts with Collagen Cross-linking Sites and Activates Lysyl Oxidase. J. Biol. Chem..

[49] Fatoux-Ardore M (2014). Large-scale investigation of Leishmania interaction networks with host extracellular matrix by surface plasmon resonance imaging. Infect. Immun..

[50] Kohfeldt E, Maurer P, Vannahme C, Timpl R (1997). Properties of the extracellular calcium binding module of the proteoglycan testican. FEBS Lett..

[51] Brizzard BL, Chubet RG, Vizard DL (1994). Immunoaffinity purification of FLAG epitope-tagged bacterial alkaline phosphatase using a novel monoclonal antibody and peptide elution. Biotechniques.

[52] Ishida T, Kinoshita K (2008). Prediction of disordered regions in proteins based on the meta approach. Bioinformatics.

[53] Dosztányi Z, Mészáros B, Simon I (2009). ANCHOR: web server for predicting protein binding regions in disordered proteins. Bioinformatics.

[54] Malhis N, Jacobson M, Gsponer J (2016). MoRFchibi SYSTEM: software tools for the identification of MoRFs in protein sequences. Nucleic Acids Res..

[55] Provencher SW, Glöckner J (1981). Estimation of globular protein secondary structure from circular dichroism. Biochemistry.

[56] Sreerama N, Woody RW (2000). Estimation of protein secondary structure from circular dichroism spectra: comparison of CONTIN, SELCON, and CDSSTR methods with an expanded reference set. Anal. Biochem..

[57] Whitmore L, Wallace BA (2008). Protein secondary structure analyses from circular dichroism spectroscopy: methods and reference databases. Biopolymers.

[58] Pernot P (2013). Upgraded ESRF BM29 beamline for SAXS on macromolecules in solution. J. Synchrotron Radiat..

[59] Round A (2015). BioSAXS Sample Changer: a robotic sample changer for rapid and reliable high-throughput X-ray solution scattering experiments. Acta Crystallogr. D Biol. Crystallogr..

[60] Incardona M-F (2009). EDNA: a framework for plugin-based applications applied to X-ray experiment online data analysis. J. Synchrotron Radiat..

[61] Franke D (2017). ATSAS 2.8: a comprehensive data analysis suite for small-angle scattering from macromolecular solutions. J. Appl. Cryst..

[62] Konarev PV, Svergun DI (2015). A posteriori determination of the useful data range for small-angle scattering experiments on dilute monodisperse systems. IUCrJ.

[63] Konarev PV, Volkov VV, Sokolova AV, Koch MHJ, Svergun DI (2003). PRIMUS: a Windows PC-based system for small-angle scattering data analysis. J. Appl. Cryst..

[64] Förster S, Apostol L, Bras W (2010). Scatter: software for the analysis of nano- and mesoscale small-angle scattering. J. Appl. Cryst..

[65] Kikhney AG, Svergun DI (2015). A practical guide to small angle X-ray scattering (SAXS) of flexible and intrinsically disordered proteins. FEBS Lett..

[66] Faye C, Chautard E, Olsen BR, Ricard-Blum S (2009). The first draft of the endostatin interaction network. J. Biol. Chem..

[67] Salza R (2014). Extended interaction network of procollagen C-proteinase enhancer-1 in the extracellular matrix. Biochem. J..

[68] Ricard-Blum S (2004). Characterization of endostatin binding to heparin and heparan sulfate by surface plasmon resonance and molecular modeling: role of divalent cations. J. Biol. Chem..

[69] Shannon P (2003). Cytoscape: a software environment for integrated models of biomolecular interaction networks. Genome Res..

[70] Liwo A, Czaplewski C, Ołdziej S, Scheraga HA (2008). Computational techniques for efficient conformational sampling of proteins. Curr. Opin. Struct. Biol..

[71] Liwo A (2014). A unified coarse-grained model of biological macromolecules based on mean-field multipole-multipole interactions. J. Mol. Model..

[72] Laskowski RA (2009). PDBsum new things. Nucleic Acids Res..

[73] Hutchinson EG, Thornton JM (1990). HERA–a program to draw schematic diagrams of protein secondary structures. Proteins.

[74] Morris GM (1998). Automated docking using a Lamarckian genetic algorithm and an empirical binding free energy function. J. Comput. Chem..

[75] Pichert A (2012). Characterization of the interaction of interleukin-8 with hyaluronan, chondroitin sulfate, dermatan sulfate and their sulfated derivatives by spectroscopy and molecular modeling. Glycobiology.

[76] Kirschner KN (2008). GLYCAM06: a generalizable biomolecular force field. Carbohydrates. J. Comput. Chem..

[77] Case, D. A. *et al*. AMBER 2017. *University of California*, *San Francisco* (2017).

[78] Ester M, Kriegel H, Sander J, Xu X (1996). A density-based algorithm for discovering clusters a density-based algorithm for discovering clusters in large spatial databases with noise. KDD.

[79] Samsonov SA, Gehrcke J-P, Pisabarro MT (2014). Flexibility and explicit solvent in molecular-dynamics-based docking of protein-glycosaminoglycan systems. J. Chem. Inf. Model..

[80] Nivedha AK, Makeneni S, Foley BL, Tessier MB, Woods RJ (2014). Importance of ligand conformational energies in carbohydrate docking: Sorting the wheat from the chaff. J. Comput. Chem..

[81] Humphrey W, Dalke A, Schulten K (1996). VMD: visual molecular dynamics. J. Mol. Graph..

[82] Gandhi NS, Mancera RL (2009). Free energy calculations of glycosaminoglycan-protein interactions. Glycobiology.

[83] Homeyer N, Gohlke H (2012). Free Energy Calculations by the Molecular Mechanics Poisson-Boltzmann Surface Area Method. Mol. Inform..

[84] Naba A (2016). The extracellular matrix: Tools and insights for the “omics” era. Matrix Biol..

