# Supplementary material for: Insights into the structure and dynamics of lysyl oxidase propeptide, a flexible protein with numerous partners
Source: Sci Rep. 2018 Aug 6;8:11768. doi: 10.1038/s41598-018-30190-6 (PMC6078952; doi:10.1038/s41598-018-30190-6)
Supplement: Supplementary file 1 — Supplementary material [file 41598_2018_30190_MOESM1_ESM.pdf]

Insights into the structure and dynamics of lysyl oxidase propeptide, a flexible protein with numerous partners

Sylvain D. Vallet, Adriana E. Miele, Urszula Uciechowska-Kaczmarzyk, Adam Liwo, Bertrand Duclos, Sergey A. Samsonov, Sylvie Ricard-Blum

SUPPLEMENTARY FIGURES

Supplementary Figure 1. Deglycosylation of the propeptide of human lysyl oxidase by PNGase F.

Supplementary Figure 2. Analysis of the propeptide of human lysyl oxidase by circular dichroism with and without heparin.

Supplementary Figure 3. Analysis of the propeptide of human lysyl oxidase by SEC-SAXS.

Supplementary Figure 4. Models the propeptide of human lysyl oxidase and SAXS data

Supplementary Figure 5. Topology of the models of the propeptide of human lysyl oxidase generated with PROMOTIF software.

Supplementary Figure 6. Arginine residues of the propeptide of human lysyl oxidase contributing to the binding of heparin hexasaccharide.

Supplementary Figure 7. Surface Plasmon Resonance imaging (SPRi) binding assays.

Supplementary Figure 8. Surface Plasmon Resonance (SPR) and Bio-Layer Interferometry (BLI) binding assays. **a**) Sensorgrams collected by SPR. **b**) Kinetic analysis performed by SPR for plasminogen (62.5-1000 nM) and tropoelastin (2.5-40 µM) and by BLI for anastellin. Experimental (black) and fitted (red) data.

SUPPLEMENTARY TABLES

Supplementary Table 1. Changes in the radius of gyration (R_g) of LOX-PP during MD simulation in the presence of a heparin hexasaccharide.

Supplementary Table 2. Changes in the radius of gyration (R_g) of LOX-PP during MD simulation in the absence of a heparin hexasaccharide.

Supplementary Table 3. Secondary structure of LOX-PP/heparin hexasaccharide complexes in the MD trajectories after 10 ns and 20 ns of simulation based on DSSP analysis obtained from the cpptraj module of the AMBER package.

Supplementary Table 4. Free energy contribution (kcal/mol) of arginine and negatively charged residues for the five models of LOX-PP complexed with heparin hexasaccharide.

Supplementary Table 5. List of LOX-PP partners reported in the literature with the detection methods and the PMIDs reporting the interactions.

Supplementary Table 6. List of biomolecules tested in binding assays performed by Surface Plasmon Resonance (SPR) imaging, SPR and Bio-Layer Interferometry (BLI).

Supplementary Table 7. List of LOX-PP partners identified in this study by SPRi, SPR, and BLI binding assays. Negative interactions are also indicated.

SUPPLEMENTARY METHOD: COARSE-GRAINED SIMULATIONS

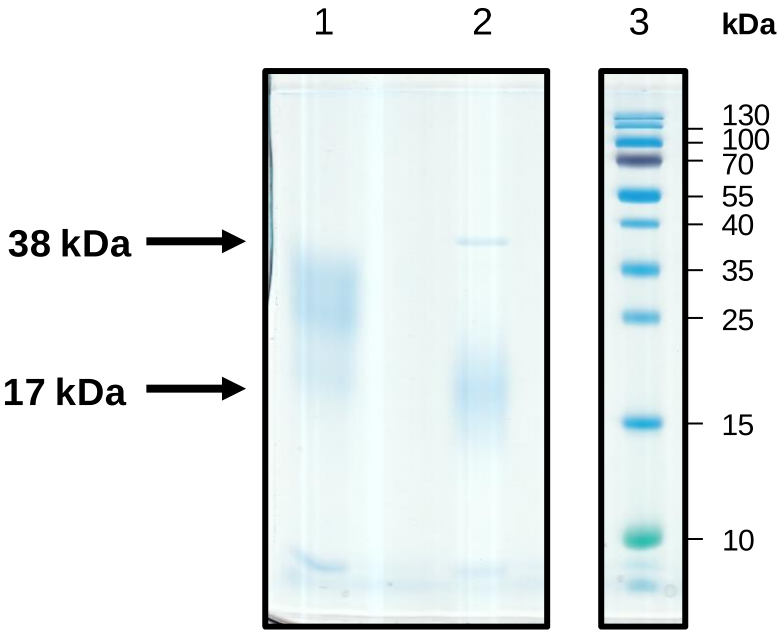

Supplementary Figure 1. Deglycosylation of the propeptide of human lysyl oxidase by PNGase F. LOX-PP (8.2 µg) incubated without (lane 1) or with PNGase F (lane 2) as described below. Lane 3: molecular weight markers.

18 µl of LOX-PP solution at 0.92 mg/ml in HBS (16.6 µg) was incubated at 55°C for 10 min with 2 µl 400 mM DTT, and then at 37°C for 1 h with 2 µl PNGase F according to the manufacturer's instructions (New England's Biolab, P0706). The reaction medium was then precipitated with 4 volumes of acetone at -20 °C for 1 h and centrifuged 10 min at 16 000 × *g*. Finally, the pellet was resuspended in 20 µl of reducing loading buffer and analyzed by SDS-PAGE on a 15 % polyacrylamide gel.

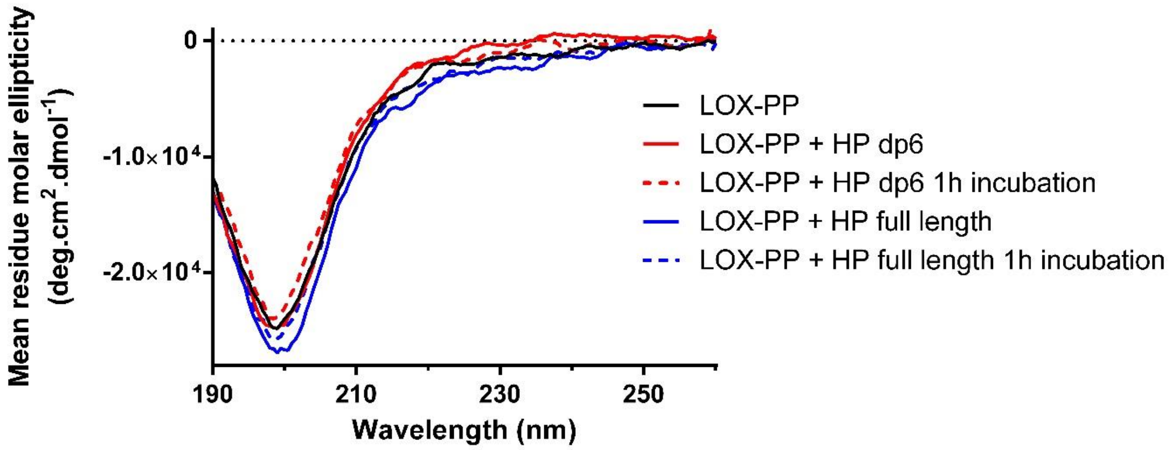

Supplementary Figure 2. Analysis of the propeptide of human lysyl oxidase by circular dichroism with and without heparin. Averaged circular dichroism spectra (n=5) of LOX-PP at 2 µM (60 µg/ml) in 10 mM potassium phosphate pH 7.4. LOX-PP was analyzed in absence and presence of 2 µM of heparin hexasaccharide (dp6) or full-length heparin immediately after mixing or after one-hour incubation at room temperature.

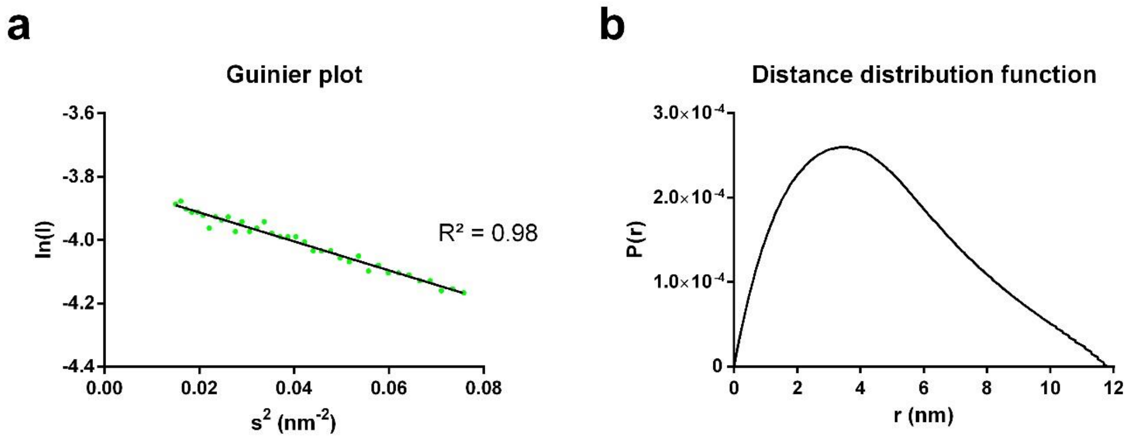

Supplementary Figure 3. **Analysis of the propeptide of human lysyl oxidase by SEC-SAXS**. SEC-SAXS experiments were performed on the SWING beamline (French National Synchrotron Facility SOLEIL, Saint-Aubin, France, 20170906). 50 µl of LOX-PP (219 µM) were injected in HBS on a Superdex 200 Increase 5/150 GL column at a flow rate of 0.2 ml/min. The parameters used for SAXS analysis were the following: acquisition 1 frame/s, detector distance: 2.087 m, wavelength: 1.033 Å, detector: Eiger 4M (beam center 1020 × 646 pixel). Foxtrot was used for data reduction and frame selection. Buffer spectra collected before the void volume were subtracted from LOX-PP spectra using CHROMIXS (*Panjkovich and Svergun, 2018*). Useful data range was defined by SHANUM (*Konarev and Svergun, 2015*). PRIMUS (*Konarev et al., 2003*) was used to calculate **a**) the radius of gyration of LOX-PP using the Guinier plot with a sR_g limit < 1.02 and **b**) D_{max} using the distance distribution function.

Konarev, P. V. & Svergun, D. I. A posteriori determination of the useful data range for small-angle scattering experiments on dilute monodisperse systems. IUCrJ 2, 352-360 (2015).

Konarev, P. V., Volkov, V. V., Sokolova, A. V., Koch, M. H. J. & Svergun, D. I. PRIMUS: a Windows PC-based system for small-angle scattering data analysis. J. Appl. Cryst. 36, 1277-1282 (2003).

Panjkovich, A. & Svergun, D. I. CHROMIXS: automatic and interactive analysis of chromatography-coupled small-angle X-ray scattering data. Bioinformatics 34, 1944-1946 (2018).

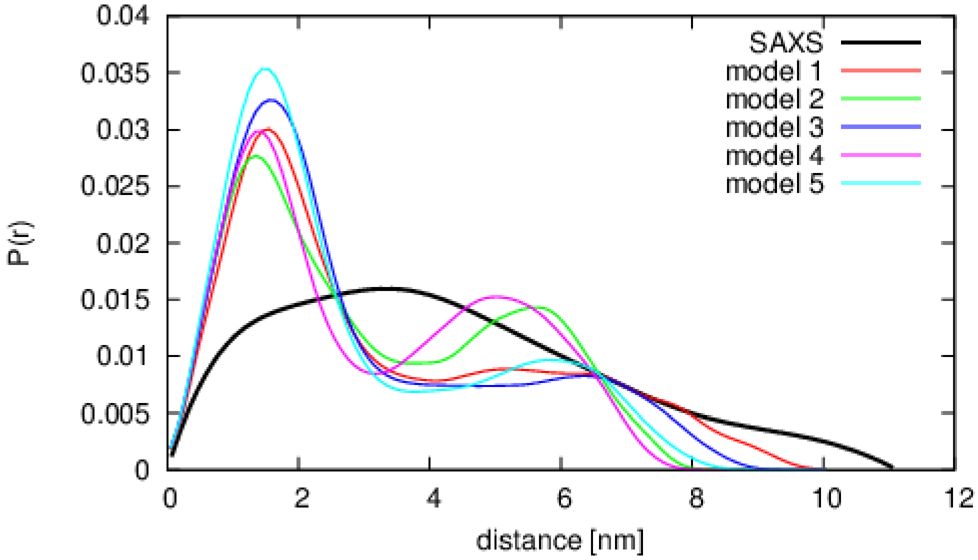

Supplementary Figure 4. **Models the propeptide of human lysyl oxidase and SAXS data**. Gaussian-smoothed C_{α}-distance distribution from SAXS data for LOX-PP and for the five models predicted using the UNRES force field.

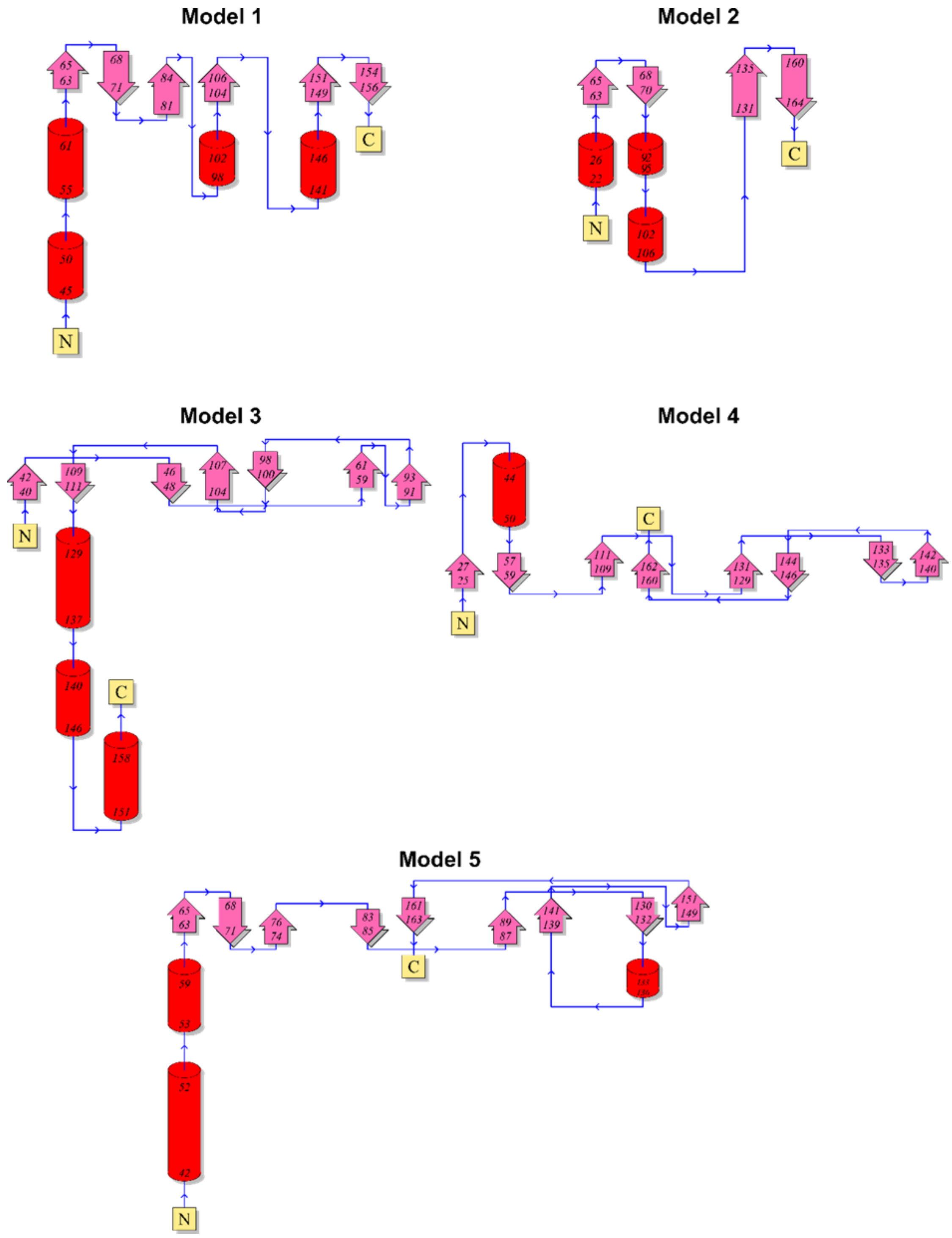

Supplementary Figure 5. **Topology of the models of the propeptide of human lysyl oxidase generated with PROMOTIF software.**

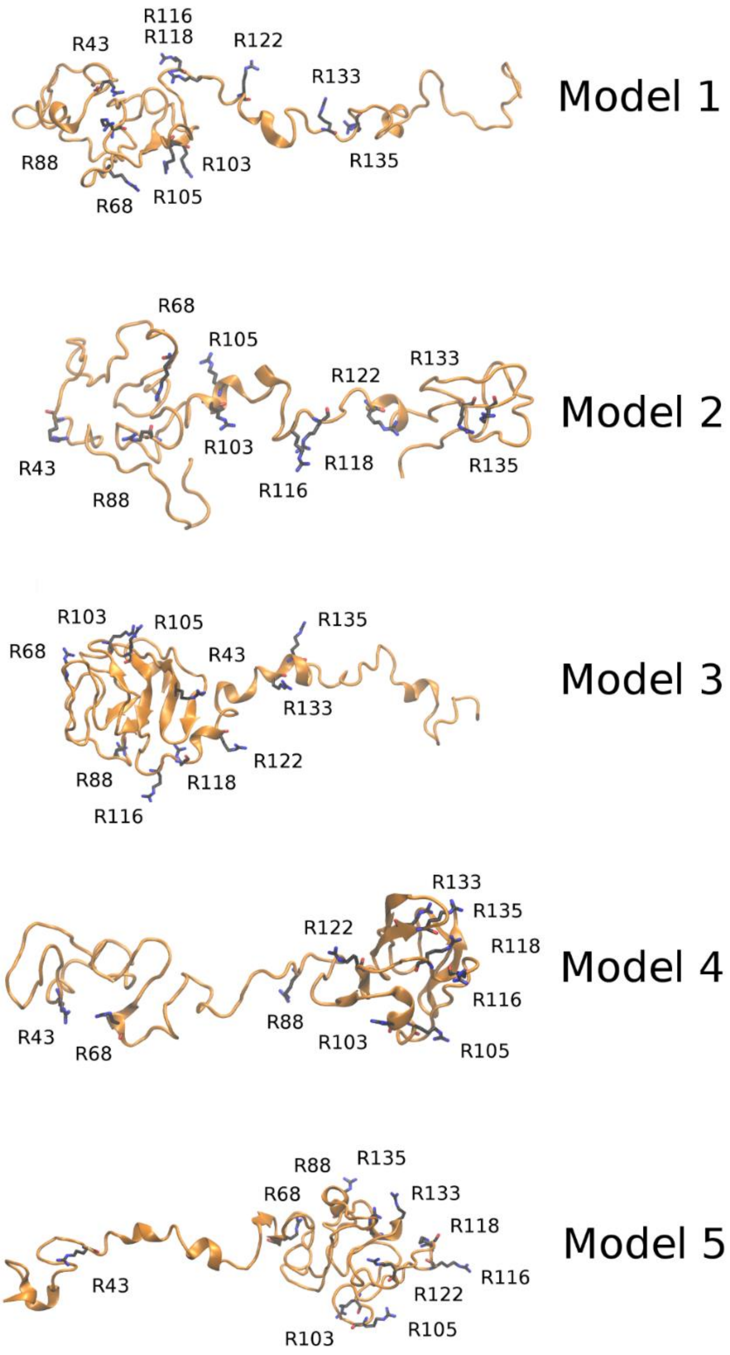

Supplementary Figure 6. **Arginine residues of the propeptide of human lysyl oxidase contributing to the binding of heparin hexasaccharide**. Visualization of the ten arginine residues (sticks) predicted to contribute the most to the heparin hexasaccharide binding according to molecular mechanics energies combined with the generalized Born and surface area continuum (MM-GBSA) solvation (free energy per residue).

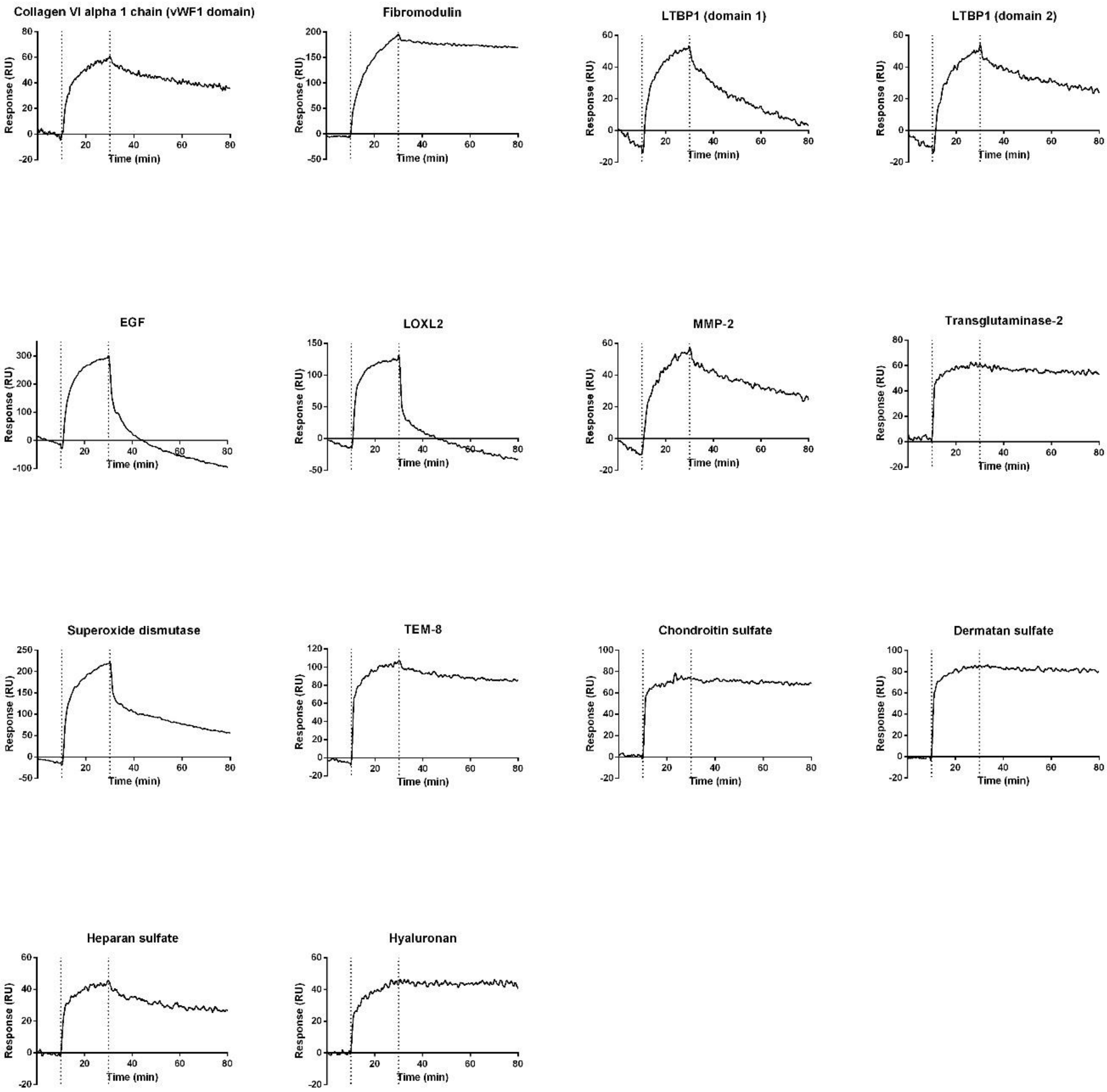

Supplementary Figure 7. **Surface Plasmon Resonance imaging (SPRi) binding assays**. The partners of the propeptide of human lysyl oxidase (ligands) were spotted in triplicate over the arrays and the propeptide of human lysyl oxidase was used as the analyte. Sensorgrams were smoothed on 6 neighboring data points and adjusted to the baseline. One representative sensorgram of each triplicate is shown. The recirculation of the propeptide of lysyl oxidase over the arrays is visualized by dotted lines.

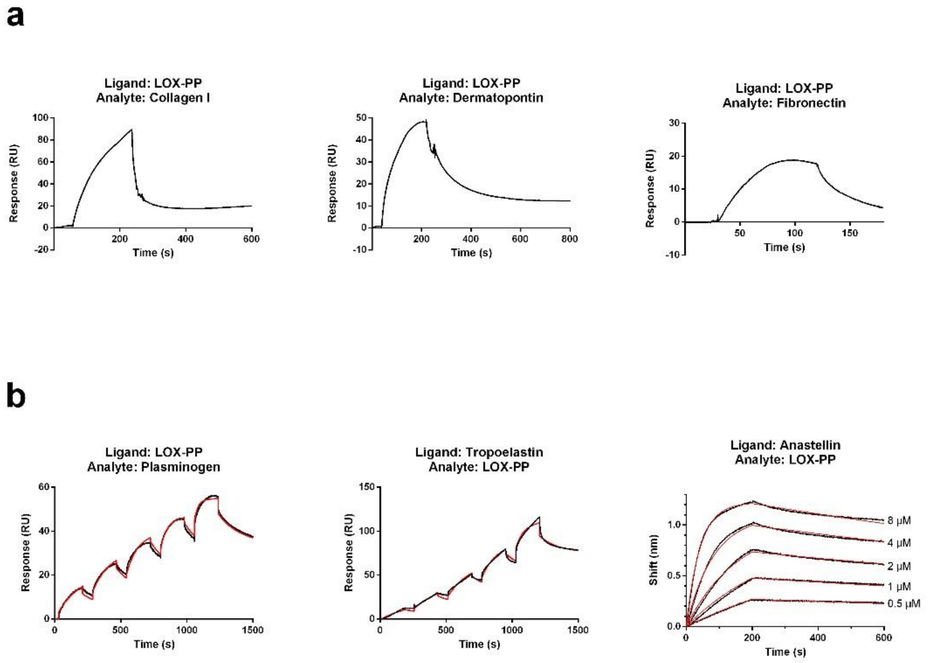

Supplementary Figure 8. Surface Plasmon Resonance (SPR) and Bio-Layer Interferometry (BLI) binding assays. **a**) Sensorgrams collected by SPR. **b**) Kinetic analysis performed by SPR for plasminogen (62.5-1000 nM) and tropoelastin (2.5-40 µM) and by BLI for anastellin. Experimental (black) and fitted (red) data.

Supplementary Table 1. Changes in the radius of gyration (R_g) of LOX-PP during MD simulation in the presence of a heparin hexasaccharide.

**Model**	**Cluster**	**R_g (nm) at 10 ns**	**R_g (nm) at 20 ns**
1	1	1.92±0.04	1.78±0.03
		2.62±0.05	2.05±0.04
		2.05±0.03	1.96±0.01
	2	2.44±0.04	2.68±0.06
		2.87±0.07	2.27±0.03
		3.21±0.06	3.11±0.05
2	1	2.25±0.05	2.50±0.08
		2.34±0.05	2.16±0.02
		2.11±0.04	2.31±0.04
3	1	2.22±0.08	2.02±0.04
		2.28±0.03	1.92±0.02
		1.91±0.03	1.77±0.03
	2	2.47±0.05	2.39±0.03
		2.09±0.09	2.15±0.05
		2.20±0.03	2.25±0.04
4	1	3.19±0.03	3.26±0.02
		2.37±0.04	1.90±0.03
		2.24±0.06	2.07±0.03
	2	2.96±0.03	2.94±0.04
		2.03±0.06	2.24±0.07
		3.05±0.05	3.30±1.00
	3	2.31±0.03	2.28±0.08
		2.16±0.05	2.17±0.04
		2.45±0.05	2.79±0.02
	4	3.21±0.05	3.38±0.08
		3.28±0.03	3.30±0.05
		3.28±0.03	2.84±0.03
5	1	2.13±0.04	2.06±0.05
		1.99±0.06	1.83±0.02
		2.25±0.04	2.12±0.06

Supplementary Table 2. Changes in the radius of gyration (R_g) of LOX-PP during MD simulation in the absence of a heparin hexasaccharide.

LOX-PP model	R_g (nm)		
	Initial	10 ns	20 ns
1	2.93	2.63 ± 0.05	2.16 ± 0.04
2	2.74	2.84 ± 0.07	3.0 ± 0.10
3	2.58	1.86 ± 0.03	1.81 ± 0.01
4	2.73	2.05 ± 0.05	2.24 ± 0.04
5	2.56	1.96 ± 0.04	1.89 ± 0.02

Supplementary Table 3. Secondary structure of LOX-PP/HP complexes in the MD trajectories after 10 ns and 20 ns of simulation based on DSSP analysis obtained from the cpptraj module of AMBER package, which considers all types of helices as α helices. (DSSP: Dictionary of Secondary Structure of Proteins)

Model	Cluster	DSSP 10 ns		DSSP 20 ns	
		α-helix (%)	β-sheet (%)	α-helix (%)	β-sheet (%)
1	1	11.5	5.4	11.5	8.2
		15.6	4.7	8.8	6.2
		6.8	8.2	15	4.7
	2	15	3.4	10.2	9.5
		10.8	10.8	15	9.5
		5.4	12.2	6.2	10.2
2	1	10.8	4.7	12.3	5.4
		12.3	5.4	12.3	8.8
		7.5	8.2	6.2	11.5
3	1	11.5	17.6	10.8	14.5
		6.8	12.3	8.8	12.3
		12.2	11.5	11.5	14.3
	2	11.5	17.6	13.2	15.6
		6.8	12.3	6.8	11.5
		12.3	11.5	10.2	15.6
4	1	8.2	8.8	11.5	8.2
		10.8	15.6	12.3	13.6
		12.3	8.2	14.3	6.2
	2	11.5	10.2	10.8	6.8
		12.3	10.8	12.3	12.3
		14.3	4.7	12.3	6.2
	3	10.8	8.8	10.2	9.5
		7.5	13.6	8.2	12.3
		6.2	14.3	7.5	13.2
	4	11.5	13.6	10.8	10.8
		4.7	7.5	6.8	15
		13.6	7.5	10.8	4.2
5	1	13.2	7.5	8.8	5.4
		8.2	8.2	10.2	8.2
		17.2	5.4	11.5	4.7

Supplementary Table 4. Free energy contribution (kcal/mol) of arginine, histidine and negatively charged residues for the five models of LOX-PP complexed with HP hexasaccharide. Mean values are obtained from all MD simulations.

Residue	Model 1	Model 2	Model 3	Model 4	Model 5
R33	-1.56	-1.33	-8.23	-0.40	-0.69
R43	-4.90	-4.45	-5.07	-0.37	-0.73
R68	-6.01	-14.96	-4.66	-0.47	-0.93
R69	-1.38	-2.88	-0.97	-0.46	-1.10
R70	-6.05	-7.22	-1.01	-0.49	-1.03
R88	-7.58	-15.01	-0.74	-0.71	-0.95
R95	-0.95	-0.89	-0.97	-0.80	-1.06
R98	-2.30	-1.00	-6.38	-0.99	-1.18
R103	-7.66	-3.10	-6.42	-5.34	-14.50
R105	-9.17	-2.11	-9.90	-2.09	-1.19
R116	-3.90	-6.60	-0.78	-15.24	-0.69
R118	-3.02	-2.14	-0.94	-12.05	-0.74
R122	-4.02	-0.84	-0.91	-1.34	-0.90
R133	-2.17	-0.73	-3.34	-3.79	-0.79
R135	-0.88	-0.66	-1.21	-6.10	-0.85
R141	-0.92	-0.63	-2.71	-2.79	-0.77
R158	-0.58	-0.59	-0.68	-0.90	-5.04
R162	-0.56	-0.80	-0.68	-1.01	-2.98
H123	-0.16	-0.05	0.04	0.05	0.05
D71	1.42	1.50	0.93	0.52	1.26
D96	1.12	0.98	1.27	0.91	1.11
D164	0.48	0.81	0.60	1.21	1.55
E34	1.07	2.56	4.18	0.37	0.65
E49	1.32	0.99	1.98	0.42	0.80
E136	0.98	0.64	1.02	2.09	0.83
E143	0.73	0.70	0.93	1.17	0.80
E150	0.62	0.64	0.69	0.85	0.88

Supplementary Table 5. List of LOX-PP partners reported in the literature with the detection methods and the PMIDs reporting the interactions.

**LOX-PP partners reported in the literature**	**Interaction detection methods**	**PMIDs**
Apoptotic protease-activating factor 1	Pull down	21536655
Collagen I alpha1 chain	Two-hybrid	21690299
Collagen I alpha2 chain	Two-hybrid	21690299
Collagen III alpha1 chain	Two-hybrid	21690299
Double-strand break repair protein MRE11	Pull down, co-immunoprecipitation	24882580
Fibronectin	Two-hybrid	21690299
Fibulin-4	Co-immunoprecipitation	19855011
Heparin (6 and 16 kDa)	Bio-layer interferometry	DOI: 10.1039/9781788010283-00398
Heat shock 70 kDa protein 1A	Pull down, co-immunoprecipitation	21536655
Heat shock 70 kDa protein 1B	Pull down, co-immunoprecipitation	21536655
Heat shock cognate 71 kDa protein	Pull down	21536655
MMP-2	Enzymatic assay	8636146
MMP-10	iTRAQ-TAILS**	24281761
Protein UXT	Two-hybrid	28106301
RAF proto-oncogene serine/threonine-protein kinase	Pull down	21536655
Receptor-type tyrosine-protein phosphatase kappa	Two hybrid, co-immunoprecipitation	21690299
SH3 domain-containing kinase-binding protein 1	Co-immunoprecipitation	24167568
Tropoelastin	Two-hybrid, far Western blotting	16251195
Tubulin alpha-3C/D chain	Pull down	21536655
Tubulin beta chain	Pull down	21536655

** iTRAQ-TAILS: Isobaric tags for relative and absolute quantitation -Terminal amine isotopic labeling of substrates.

Supplementary Table 6. List of biomolecules tested in binding assays performed by Surface Plasmon Resonance (SPR) imaging, SPR and Bio-Layer Interferometry (BLI). ChEBI: Chemical Entities of Biological Interest, CPX: Complex Portal identifier, PRO_features are from UniProtKB.

**Biomolecules tested in binding assay**s	**Biomolecule** ChEBI, UniprotKB, or Complex Portal **identifiers** (1st-last amino acid residues of the proteins and protein fragments used in binding assays	**Reference**s (commercial sources or PMID/doi)
α-synuclein	P37840	Boston Biochem, SP-480
Adiponectin	Q15848	Sigma-Aldrich, SRP4901
Aggrecan	P13608	Sigma-Aldrich, A1960
Agrin	O00468 (1260-2045)	R&D Systems, 6624-AG
Angiopoietin-like protein 4 (AngPTL-4), mutant Lys163Ala, Arg164Ala	Q9BY76 (26-406)	R&D Systems, 4487-AN
Anthrax toxin receptor 1 (Tumor endothelial marker 8, TEM-8)	Q9H6X2-2 (1-368)	Abnova, H00084168-P01
β-2 microglobulin	P61769	Sigma-Aldrich, M4890
Biglycan	P21809	Sigma-Aldrich, B8041
Brevican	Q96GW7 (23-911)	R&D Systems, 4009-BC
Calreticulin	P27797	Abcam, ab91577
Catalase	P00432	Sigma-Aldrich, C9322
Chondroitin sulfate	CHEBI:37397	Sigma-Aldrich, C8529
Coagulation factor X	P00742	Sigma-Aldrich, 233282
Collagen I	CPX-1650	Sigma-Aldrich, C7774
Collagen II	CPX-3105	Sigma-Aldrich, C1188
Collagen III	CPX-1714	Sigma-Aldrich, C4407
Collagen IV	CPX-1723	Sigma-Aldrich, C7521
Collagen V	CPX-1727	Sigma-Aldrich, C3657

Collagen VI	CPX-1736	GeneTex, GTX27538
Collagen VI (vWF1 domain)	P12109 (20-256)	Expressed in the laboratory (PMID 24117177)
Collagen VI (vWF2-3 domain)	P12109 (593-1028)	Expressed in the laboratory (PMID 24117177, 28106549)
Collagen XIII ectodomain	CPX-1754 (62-717)	Expressed in the laboratory (PMID: 28106549)
Collagen XVII ectodomain	CPX-1758 (490-1497)	Expressed in the laboratory (PMID: 20861347, 24117177, 28106549)
Collagen XVIII (NC1 domain)	CPX-1759 (1443-1754)	Expressed in the laboratory (PMID: 10449407, 14585835, 19542224, 19502598, 24478075, 24117177, 28106549)
Collagen XXIII ectodomain	CPX-1764 (111-540)	Expressed in the laboratory
Collagen XXV ectodomain	CPX-1766 (113-654)	Expressed in the laboratory
Connective tissue growth factor (CTGF)	P29279	RayBiotech, 228-10290-2
Discoidin domain-containing receptor 1 ectodomain (DDR1)	Q5ST11 (21-416)	R&D Systems, 2396-DR
Discoidin domain-containing receptor 2 ectodomain (DDR2)	Q16832 (24-399)	R&D Systems, 2538-DR
Decorin	P07585 (17-359)	R&D Systems, 143-DE
Dermatan sulfate	CHEBI:18376	Sigma-Aldrich, C3788
Dermatopontin	Q07507	Abcam, ab158310
Extracellular matrix protein 1 (ECM1)	Q16610 (20-540)	R&D Systems, 3937-EC
Endostatin (expressed in *Pichia pastoris*)	P39060 (PRO_0000005794)	Sigma-Aldrich, E8154

Endostatin (expressed in HEK293 cells)	P39060 (PRO_0000005794)	Expressed in the laboratory (PMID: 14585835, 19542224, 19502598, 24117177, 24478075, 28106549, DOI: 10.1039/9781788010283-00398)
Endostatin, mutant Asp1675Asn	P39060 (PRO_0000005794)	Expressed in the laboratory (PMID: 19502598)
Endostatin, mutant Arg1598Ala, Arg1710Ala	P39060 (PRO_0000005794)	Expressed in the laboratory (PMID: 19502598)
Enolase		Sigma-Aldrich, E6126
Epidermal growth factor (EGF)	P01133 (PRO_0000007541)	Sigma-Aldrich, E9644
Epidermal growth factor receptor (EGFR)	P00533 (25-647)	RayBiotech, 228-10367-2
Epigen	Q6UW88	Sigma-Aldrich, SRP4969
Fibroblast growth factor-2	P09038 (PRO_0000008933)	PromoKine, C60240
Fibromodulin	P13605	Sigma-Aldrich, F6921
Fibronectin (cellular)	P02751	Sigma-Aldrich, F2518
Fibronectin (plasma)	P02751	Sigma-Aldrich, F2006
Fibronectin fragment III_1-C (anastellin)	P02751 (PRO_0000390479, 631-702)	Sigma-Aldrich, F3542
Fibulin 4	O95967 (26-443)	USCN, RPF421HU01
Glypican 1	P35052 (24-530)	R&D Systems, 4519-GP
Glypican 2	Q8N158 (18-553)	R&D Systems, 2304-GP
Glypican 3	P51654	R&D Systems, 2119-GP
Glypican 5	P78333 (25-554)	R&D Systems, 2607-G5
Glypican 6	Q9Y625 (24-355)	R&D Systems, 2845-GP
Heparan sulfate	CHEBI:28815	Celsus Lab, HO-3105
Heparin (high molecular weight)	CHEBI:28304	Sigma-Aldrich, H3393
Heparin (low molecular weight, 3 kDa)	CHEBI:28304	Sigma-Aldrich, H3400
Hyaluronan	CHEBI:16336	Acros Organics, 25177

Hyaluronan (25-75 kDa)	CHEBI:16336	Sigma-Aldrich, S0326
Hyaluronidase-1	Q12794	R&D Systems, 7358-GH
Integrin α4β1 ectodomain	CPX-1802	R&D Systems, 5668-A4
Integrin α5β1 ectodomain	CPX-1794	R&D Systems, 3230-A5
Integrin αvβ3 ectodomain	CPX-1795	R&D Systems, 3050-AV
Integrin αvβ5 ectodomain	CPX-1796	R&D Systems, 2528-AV
Laminin-111	CPX-3008	Sigma-Aldrich, L2020
Latent transforming growth factor beta-1 (TGFβ1)	P01137	Sino Biological, 10804-H08H
Latent transforming growth factor β binding protein 1 (4-7 EGF-like domains)	Q14766 (873-1037)	Generous gift from Dr. L. Perrin-Cocon and Dr. V. Lotteau (International Center for Infectiology Research, Lyon, France) (PMID: 24117177)
Latent transforming growth factor β binding protein 1 (9-14 EGF-like domains)	Q14766 (1079-1328)	Generous gift from Dr. L. Perrin-Cocon and Dr. V. Lotteau (International Center for Infectiology Research, Lyon, France) (PMID: 24117177)
Leukocyte-associated immunoglobulin-like receptor-1 (LAIR1)	Q6GTX8 (22-163)	R&D Systems, 2664-LR
Lysyl oxidase propeptide (LOX-PP) (Pro24Leu)	P28300 (PRO_0000018520)	Expressed in the laboratory (DOI: 10.1039/9781788010283-00398)
Lysyl oxidase homolog 2 (LOXL2)	Q9Y4K0	R&D Systems, 2639-AO
Lysyl oxidase homolog 3 (LOXL3)	P58215	R&D Systems, 6069-AO
Lumican	P51884 (19-338)	R&D Systems, 2846-LU
Macrophage receptor MARCO	Q9UEW3 (79-520)	R&D Systems, 7586-MA
Matrix metalloproteinase-2 (MMP-2)	P08253 (30-660)	R&D Systems, 902-MPN
Neurexin-1 β	P58400 (51-363)	R&D Systems, 5268-NX
Neurocan	P55066 (23-637)	R&D Systems, 5800-NC
Neuroglycan C	O95196 (31-420)	R&D Systems, 5685-NG

Neuropilin-1 (NRP1) ectodomain	O14786, 23-815	Expressed in the laboratory (PMID, 28106549)
Osteonectin (SPARC)	P09486	Immundiagnostik AG, A4225AG.1
Osteopontin (with bovine serum albumin)	P10451	Sigma-Aldrich, O4264
Osteoprotegerin	O00300 (22-401)	R&D Systems, 185-OS
Procollagen C-proteinase enhancer 1 (PCPE-1)	Q15113	Generous gift from Dr E. Kessler (Goldschleger Eye Research Institute, Tel Aviv University Faculty of Medicine Sheba Medical Center, Tel-Hashomer Israel) (PMID: 15834133, 24117177, 28106549)
Platelet-derived growth factor receptor alpha (PDGFRα)	P16234 (24-524)	R&D Systems, 322-PR
Platelet-derived growth factor receptor beta (PDGFRβ), mutant Glu241Asp	P09619 (33-530)	R&D Systems, 385-PR
Periostin	Q15063 (22-836)	R&D Systems, 3548-F2
Perlecan	Q05793	Sigma-Aldrich, H4777
Plasminogen	P00747	R&D Systems, 1939-SE
Reelin	Q60841 (1221-2661)	R&D Systems, 3820-MR
Superoxide dismutase	Q4Q597	Expressed in the laboratory
Syndecan-1 ectodomain	P18827 (18-251)	R&D Systems, 2780-SD
Syndecan-2 ectodomain	P34741 (19-144)	R&D Systems, 2965-SD
Syndecan-3 ectodomain	O75056 (48-383)	R&D Systems, 3539-SD
Syndecan-4 ectodomain	P31431 (19-145)	R&D Systems, 2918-SD
Thrombospondin-1 (TSP-1)	CPX-1785	Immundiagnostik AG, AW1011AG.1
Transglutaminase-2 (TG-2) (guinea pig)	P08587	Sigma-Aldrich, T5398
Transglutaminase-2 (TG-2) (human)	P21980	Immundiagnostik AG, AK3010AG.1
Transglutaminase-2 (TG-2) (human)	P21980	Zedira, T022

Transglutaminase-2 (TG-2), mutant Cys277Ser	P21980	Zedira, T018
Tropoelastin	P15502	Sigma-Aldrich, T0706
Tropomyosin		Sigma-Aldrich, T2400
Tumor necrosis factor α (TNFα)	P01375 (77-233)	R&D Systems, 210-TA
Vascular endothelial growth factor (VEGF)	CPX-1977	Sigma-Aldrich, V7259
Vascular endothelial growth factor receptor-2 (VEGFR-2)	P35968 (20-764)	R&D Systems, 357-KD
Vitronectin	P04004	R&D Systems, 2349-VN

Supplementary Table 7. List of LOX-PP partners identified in this study by SPRi, SPR, and BLI binding assays. Negative interactions are also indicated (nt: not tested)

**Biomolecules tested in binding assays** (no: no binding, yes: binding, nt: not tested)	**Interaction detection method**		
	**SPRi (Surface Plasmon Resonance imaging)**	**SPR (Surface Plasmon Resonance)**	**BLI (Bio-Layer Interferometry)**
α-synuclein	no	nt	nt
Adiponectin	no	nt	nt
Aggrecan	no	nt	nt
Agrin	no	nt	nt
Angiopoietin-like protein 4 (AngPTL-4), mutant Lys163Ala, Arg164Ala	no	nt	nt
Anthrax toxin receptor 1 (Tumor endothelial marker 8, TEM-8)	yes	nt	nt
β-2 microglobulin	no	nt	nt
Biglycan	no	nt	nt
Brevican	no	nt	nt
Calreticulin	no	nt	nt
Catalase	no	nt	nt
Chondroitin sulfate	yes	nt	nt
Coagulation factor X	no	nt	nt
Collagen I	yes	yes	nt
Collagen II	no	nt	nt
Collagen III	no	nt	nt
Collagen IV	no	nt	nt

Collagen V	no	nt	nt
Collagen VI	no	nt	nt
Collagen VI (vWF1 domain)	yes	nt	nt
Collagen VI (vWF2-3 domain)	no	nt	nt
Collagen XIII ectodomain	no	nt	nt
Collagen XVII ectodomain	no	nt	nt
Collagen XVIII (NC1 domain)	no	nt	nt
Collagen XXIII ectodomain	no	nt	nt
Collagen XXV ectodomain	no	nt	nt
Connective tissue growth factor (CTGF)	no	nt	nt
Discoidin domain-containing receptor 1 ectodomain (DDR1)	no	nt	nt
Discoidin domain-containing receptor 2 ectodomain (DDR2)	no	nt	nt
Decorin	no	nt	nt
Dermatan sulfate	yes	nt	nt
Dermatopontin	yes	yes	nt
Extracellular matrix protein 1 (ECM1)	no	nt	nt
Endostatin (expressed in *Pichia pastoris*)	no	nt	nt

Endostatin (expressed in HEK293 cells)	no	nt	nt
Endostatin, mutant Asp1675Asn	no	nt	nt
Endostatin, mutant Arg1598Ala, Arg1710Ala	no	nt	nt
Enolase	no	nt	nt
Epidermal growth factor (EGF)	yes	yes	nt
Epidermal growth factor receptor (EGFR)	no	nt	nt
Epigen	no	nt	nt
Fibroblast growth factor-2	no	nt	nt
Fibromodulin	yes	nt	nt
Fibronectin (cellular)	yes	yes	nt
Fibronectin (plasma)	no	nt	nt
Fibronectin fragment III_1-C (anastellin)	yes	yes	yes
Fibulin 4	no	nt	nt
Glypican 1	no	nt	nt
Glypican 2	no	nt	nt
Glypican 3	no	nt	nt
Glypican 5	no	nt	nt
Glypican 6	no	nt	nt
Heparan sulfate	yes	nt	nt
Heparin (high molecular weight)	yes	yes	nt
Heparin (low molecular weight, 3 kDa)	yes	nt	nt
Hyaluronan	yes	nt	nt
Hyaluronan (25-75 kDa)	yes	nt	nt
Hyaluronidase-1	no	nt	nt
Integrin $\alpha4\beta1$ ectodomain	no	nt	nt
Integrin $\alpha5\beta1$ ectodomain	no	nt	nt

Integrin αvβ3 ectodomain	no	nt	nt
Integrin αvβ5 ectodomain	no	nt	nt
Laminin-111	no	nt	nt
Latent transforming growth factor beta-1 (TGFβ1)	no	nt	nt
Latent transforming growth factor β binding protein 1 (4-7 EGF-like domains)	yes	nt	nt
Latent transforming growth factor β binding protein 1 (9-14 EGF-like domains)	yes	nt	nt
Leukocyte-associated immunoglobulin-like receptor-1 (LAIR1)	no	nt	nt
Lysyl oxidase propeptide (LOX-PP) (Pro24Leu)	no	nt	nt
Lysyl oxidase homolog 2 (LOXL2)	yes	nt	nt
Lysyl oxidase homolog 3 (LOXL3)	no	nt	nt
Lumican	no	nt	nt
Macrophage receptor MARCO	no	nt	nt
Matrix metalloproteinase-2 (MMP-2)	yes	nt	nt
Neurexin-1 β	no	nt	nt
Neurocan	no	nt	nt
Neuroglycan C	no	nt	nt
Neuropilin-1 (NRP1) ectodomain	no	nt	nt
Osteonectin (SPARC)	no	nt	nt
Osteopontin (with bovine serum albumin)	no	nt	nt
Osteoprotegerin	no	nt	nt
Procollagen C-proteinase enhancer 1 (PCPE-1)	no	nt	nt
Platelet-derived growth factor receptor alpha (PDGFRα)	no	nt	nt

Platelet-derived growth factor receptor beta (PDGFRβ), mutant Glu241Asp	no	nt	nt
Periostin	no	nt	nt
Perlecan	no	nt	nt
Plasminogen	yes	yes	nt
Reelin	no	nt	nt
Superoxide dismutase	yes	nt	nt
Syndecan-1 ectodomain	no	nt	nt
Syndecan-2 ectodomain	no	nt	nt
Syndecan-3 ectodomain	no	nt	nt
Syndecan-4 ectodomain	no	nt	nt
Thrombospondine-1 (TSP-1)	no	nt	nt
Transglutaminase-2 (TG-2) (guinea pig)	no	nt	nt
Transglutaminase-2 (TG-2) (human)	yes	yes	nt
Transglutaminase-2 (TG-2) (human)	no	nt	nt
Transglutaminase-2 (TG-2), mutant Cys277Ser	yes	nt	nt
Tropoelastin	no	yes	nt
Tropomyosin	no	nt	nt
Tumor necrosis factor α (TNFα)	no	nt	nt
Vascular endothelial growth factor (VEGF)	no	nt	nt
Vascular endothelial growth factor receptor-2 (VEGFR-2)	no	nt	nt
Vitronectin	no	nt	nt

SUPPLEMENTARY METHOD: COARSE-GRAINED SIMULATIONS

We performed multiplexed replica exchange molecular dynamics simulations[1] using the coarse grained UNRES force field[2–4] with restraints derived from the present SAXS experiments, which were enabled in our recent work[5]. The coarse-grained UNRES model[2,3] is a reduced model of polypeptide chains, in which a polypeptide chain is represented by a sequence of α-carbon (C_α) atoms linked by virtual bonds with attached united side chains and united peptide group. United peptide groups and united side chains serve as interaction sites. The UNRES force field has been derived as a Restricted Free Energy (RFE) function of an all-atom polypeptide chain and the surrounding solvent, where the all-atom energy function is averaged over the degrees of freedom that are reduced when passing from the all-atom to the simplified coarse-grained system[6,7]. The RFE is further decomposed into factors derived from interactions within and between united interaction sites. Expansion of the factors into generalized Kubo's cumulants enable to derive approximate analytical expressions for the respective terms, including the multibody or correlation terms, which are derived in other force fields from structural databases or on a heuristic basis[8]. In this work, we used the latest variant of UNRES calibrated with a set of 7 proteins[4] and the maximum-likelihood method developed in our laboratory[9]. The pseudo-energy function U used for this approach is given by equation 1 where the U_{UNRES} is U_{UNRES} energy function, V_{templ} the template-restraint penalty term, V_{SAXS} SAXS-restrained term, w_{templ} and w_{SAXS} are the weights of the respective restraint terms. In our work the w_{templ} and w_{SAXS} were set to 1 and 100 respectively[5].

$$U = U_{UNRES} + w_{templ} V_{templ} + w_{SAXS} V_{SAXS} \qquad (1)$$

In order to sample conformational space more efficiently molecular dynamics method (MREMD) was applied[1,10]. In this approach several replicas of the system were simulated at different temperatures ($T_0, T_1, \ldots, T_M$) and after a defined timestep an exchange of temperatures between neighboring replicas (j=i+1) was attempted[11]. In the MREMD calculations, we run trajectories at 20 replicas, four trajectories per each temperature. The temperatures ranged from 210 to 500 K, spaced 10 K until 350 K from 350 K to 500 spaced 20 K. This range of temperatures covered the region of the folding-unfolding transition. Each trajectory consisted of 3×10^7 MD steps, and the replicas were exchanged every 2×10^4 MD steps. The temperature was controlled by the Berendsen thermostat with the coupling constant 48.9 fs[12]. The last 100 snapshots were taken into further analysis.

The obtained conformations were analyzed with weighted histogram analysis method WHAM[13]. The heat-capacity profile was calculated to obtain the temperature at which the ensemble is analyzed, T_a, taken as 20 K below the temperature of the major heat-capacity peak[14,15]. The probabilities of the conformations were calculated at T_a based on the WHAM analysis and minimum variance clustering[14,15]. Then cluster analysis was used to dissect the conformational ensembles and to identify the most populated regions of the conformational space. The ensembles were grouped into five clusters ranked from the largest and containing the most probable structure (rank 1) to the cluster with the least probable structures[15] (rank 5). The probabilities of the clusters were calculated by summing the probabilities of the conformations constituting a cluster which were, in turn, calculated at T_a based on the results of WHAM[14,15]. For each cluster, the conformation closest to the centroid was determined, and

considered representative of the entire cluster[14,15]. Finally, the five coarse-grained models were converted to all-atom structures by using the PULCHRA[16] and SCWRL[17] knowledge-based algorithms for all-atom backbone and all-atom side-chain reconstruction, respectively. The conversion algorithms use Cα and side-chain pseudoatom from the coarse-grained models and reconstruct the full backbone and side-chain structures based on the experimental data on backbone and side-chain low energy conformations available in the Protein Data Bank (https://www.rcsb.org/). The final refinement for all-atom models was carried out by performing energy minimization and short MD runs with AMBER 16[18].

References

1. Rhee, Y. M. & Pande, V. S. Multiplexed-replica exchange molecular dynamics method for protein folding simulation. *Biophys. J.* **84,** 775–786 (2003).
2. Liwo, A., Czaplewski, C., Ołdziej, S. & Scheraga, H. A. Computational techniques for efficient conformational sampling of proteins. *Curr. Opin. Struct. Biol.* **18,** 134–139 (2008).
3. Liwo, A. *et al.* A unified coarse-grained model of biological macromolecules based on mean-field multipole-multipole interactions. *J. Mol. Model.* **20,** 2306 (2014).
4. Krupa, P. *et al.* Maximum Likelihood Calibration of the UNRES Force Field for Simulation of Protein Structure and Dynamics. *J. Chem. Inf. Model.* **57,** 2364–2377 (2017).
5. Karczyńska, A. S. *et al.* Prediction of protein structure with the coarse-grained UNRES force field assisted by small X-ray scattering data and knowledge-based information. *Proteins* **86,** 228–239 (2018).
6. Liwo, A., Czaplewski, C., Pillardy, J. & Scheraga, H. A. Cumulant-based expressions for the multibody terms for the correlation between local and electrostatic interactions in the united-residue force field. *J. Chem. Phys.* **115,** 2323–2347 (2001).
7. Sieradzan, A. K., Makowski, M., Augustynowicz, A. & Liwo, A. A general method for the derivation of the functional forms of the effective energy terms in coarse-grained energy functions of polymers. I. Backbone potentials of coarse-grained polypeptide chains. *J. Chem. Phys.* **146,** 124106 (2017).
8. Kubo, R. Generalized Cumulant Expansion Method. *J. Phys. Soc. Jpn.* **17,** 1100–1120 (1962).
9. Zaborowski, B. *et al.* A Maximum-Likelihood Approach to Force-Field Calibration. *J. Chem. Inf. Model.* **55,** 2050–2070 (2015).
10. Hansmann, U. H. E. & Okamoto, Y. Comparative study of multicanonical and simulated annealing algorithms in the protein folding problem. *Physica A* **212,** 415–437 (1994).
11. Czaplewski, C., Kalinowski, S., Liwo, A. & Scheraga, H. A. Application of Multiplexed Replica Exchange Molecular Dynamics to the UNRES Force Field: Tests with alpha and alpha+beta Proteins. *J. Chem. Theory Comput.* **5,** 627–640 (2009).
12. Swope, W. C., Andersen, H. C., Berens, P. H. & Wilson, K. R. A computer simulation method for the calculation of equilibrium constants for the formation of physical clusters of molecules: Application to small water clusters. *J. Chem. Phys.* **76,** 637–649 (1982).
13. Kumar, S., Bouzida, D., Swendsen, R. H., Kollman, P. A. & Rosenberg, J. M. THE weighted histogram analysis method for free-energy calculations on biomolecules. I. The method. *J. Comput. Chem.* **13,** 1011–1021 (1992).

14. Krupa, P. *et al.* Prediction of Protein Structure by Template-Based Modeling Combined with the UNRES Force Field. *J. Chem. Inf. Model.* **55,** 1271–1281 (2015).
15. Liwo, A. *et al.* Modification and optimization of the united-residue (UNRES) potential energy function for canonical simulations. I. Temperature dependence of the effective energy function and tests of the optimization method with single training proteins. *J. Phys. Chem. B.* **111,** 260–285 (2007).
16. Rotkiewicz, P. & Skolnick, J. Fast procedure for reconstruction of full-atom protein models from reduced representations. *J. Comput. Chem.* **29,** 1460–1465 (2008).
17. Wang, Q., Canutescu, A. A. & Dunbrack, R. L. SCWRL and MolIDE: computer programs for side-chain conformation prediction and homology modeling. *Nat. Protoc.* **3,** 1832–1847 (2008).
18. Case, D. A. *et al.* AMBER 2017. *University of California, San Francisco* (2017).
